# Breaking the mold: 3D cell cultures reshaping the future of cancer research

**DOI:** 10.3389/fcell.2024.1507388

**Published:** 2024-11-26

**Authors:** Sandra Cordeiro, Beatriz B. Oliveira, Ruben Valente, Daniela Ferreira, André Luz, Pedro V. Baptista, Alexandra R. Fernandes

**Affiliations:** ^1^ UCIBIO, Departamento de Ciências da Vida, Faculdade de Ciências e Tecnologia, Universidade NOVA de Lisboa, Caparica, Portugal; ^2^ i4HB, Associate Laboratory – Institute for Health and Bioeconomy, Faculdade de Ciências e Tecnologia, Universidade NOVA de Lisboa, Caparica, Portugal

**Keywords:** 3D models, spheroids, patient-derived organoids (PDOs), chips, drug screening, personalized medicine

## Abstract

Despite extensive efforts to unravel tumor behavior and develop anticancer therapies, most treatments fail when advanced to clinical trials. The main challenge in cancer research has been the absence of predictive cancer models, accurately mimicking the tumoral processes and response to treatments. The tumor microenvironment (TME) shows several human-specific physical and chemical properties, which cannot be fully recapitulated by the conventional 2D cell cultures or the *in vivo* animal models. These limitations have driven the development of novel *in vitro* cancer models, that get one step closer to the typical features of *in vivo* systems while showing better species relevance. This review introduces the main considerations required for developing and exploiting tumor spheroids and organoids as cancer models. We also detailed their applications in drug screening and personalized medicine. Further, we show the transition of these models into novel microfluidic platforms, for improved control over physiological parameters and high-throughput screening. 3D culture models have provided key insights into tumor biology, more closely resembling the *in vivo* TME and tumor characteristics, while enabling the development of more reliable and precise anticancer therapies.

## 1 Introduction

**FIGURE 1:**
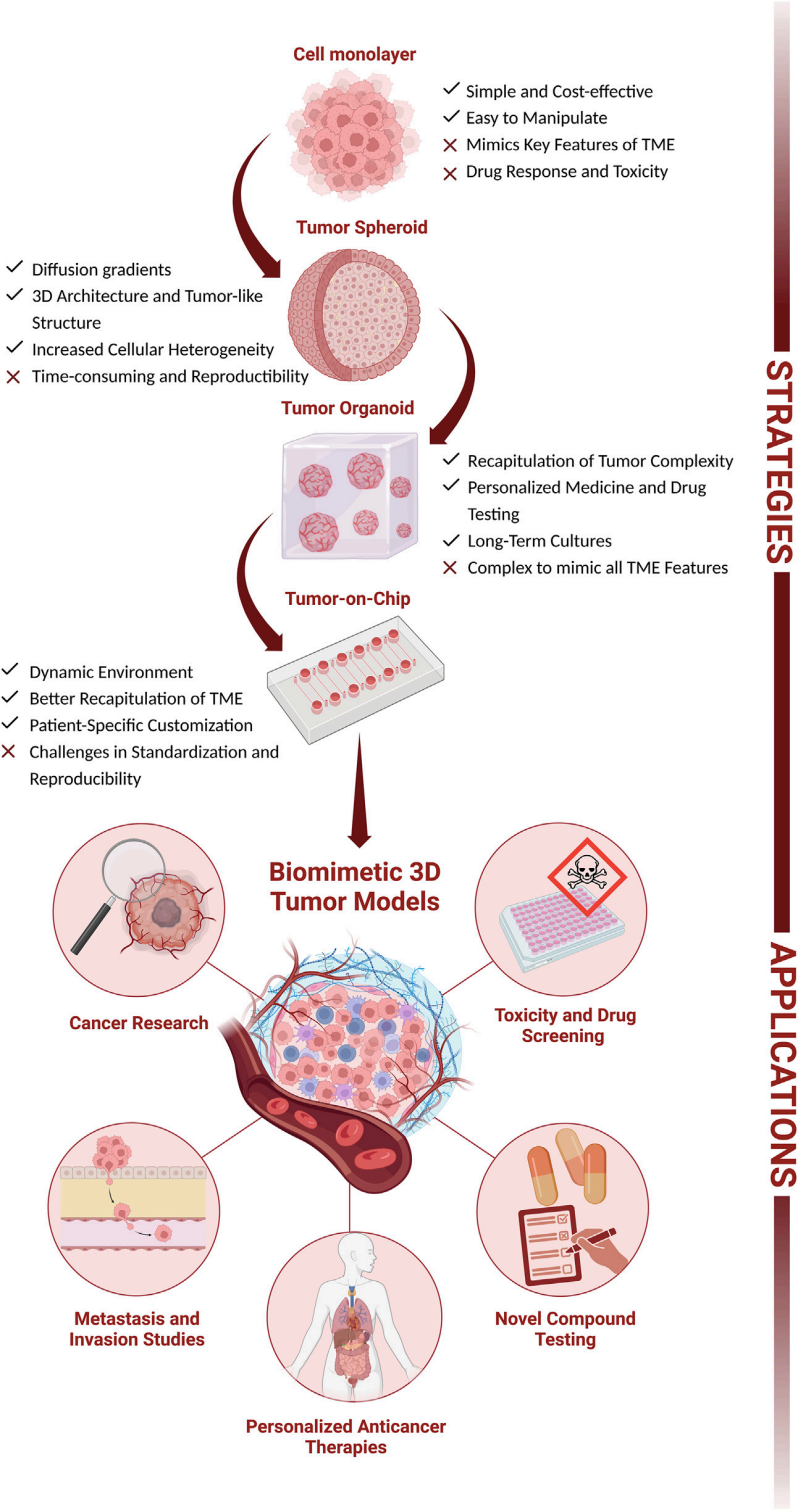
The complexity of cellular culture models: from 2D (cell monolayer) to 3D (tumor spheroids, tumor organoids, and Tumor-on-Chip). Advantages, hurdles, and main applications (cancer research, metastasis and invasion studies, personalized anticancer therapeutics, and drug development and screening). Figure created with BioRender.com.

In the past years, two-dimensional (2D) cell lines and animal models have been the cornerstone of biological research, deepening our understanding of the cellular pathways and molecular mechanisms of diseases, and as preclinical models for drug screening and toxicity testing (Drost and Clevers, 2018). Despite its wide applicability, 2D models are not capable of closely recapitulate all the characteristics found on the *in vivo* scenario, increasing the challenge of designing new target therapies (e.g., for cancer treatments). Therefore, more complex and advanced models, particularly 3D spheroids and organoids, were developed to answer the need to minimize the gap between the experimental assays and the patient’s tumor responses (Kim et al., 2020; Tang et al., 2022). Generally, 3D models offer improved resemblance to *in vivo* systems in terms of spatial organization, cellular interactions, gradient dynamics and drug responses (Barbosa et al., 2021). This review highlights the key aspects, advantages, limitations and main applications of each cell model. Further, it also explores the significant advancements that can be achieved by combining novel technologies with more sophisticated models to enhance the success of personalized anticancer therapies (Figure 1).

**FIGURE 2:**
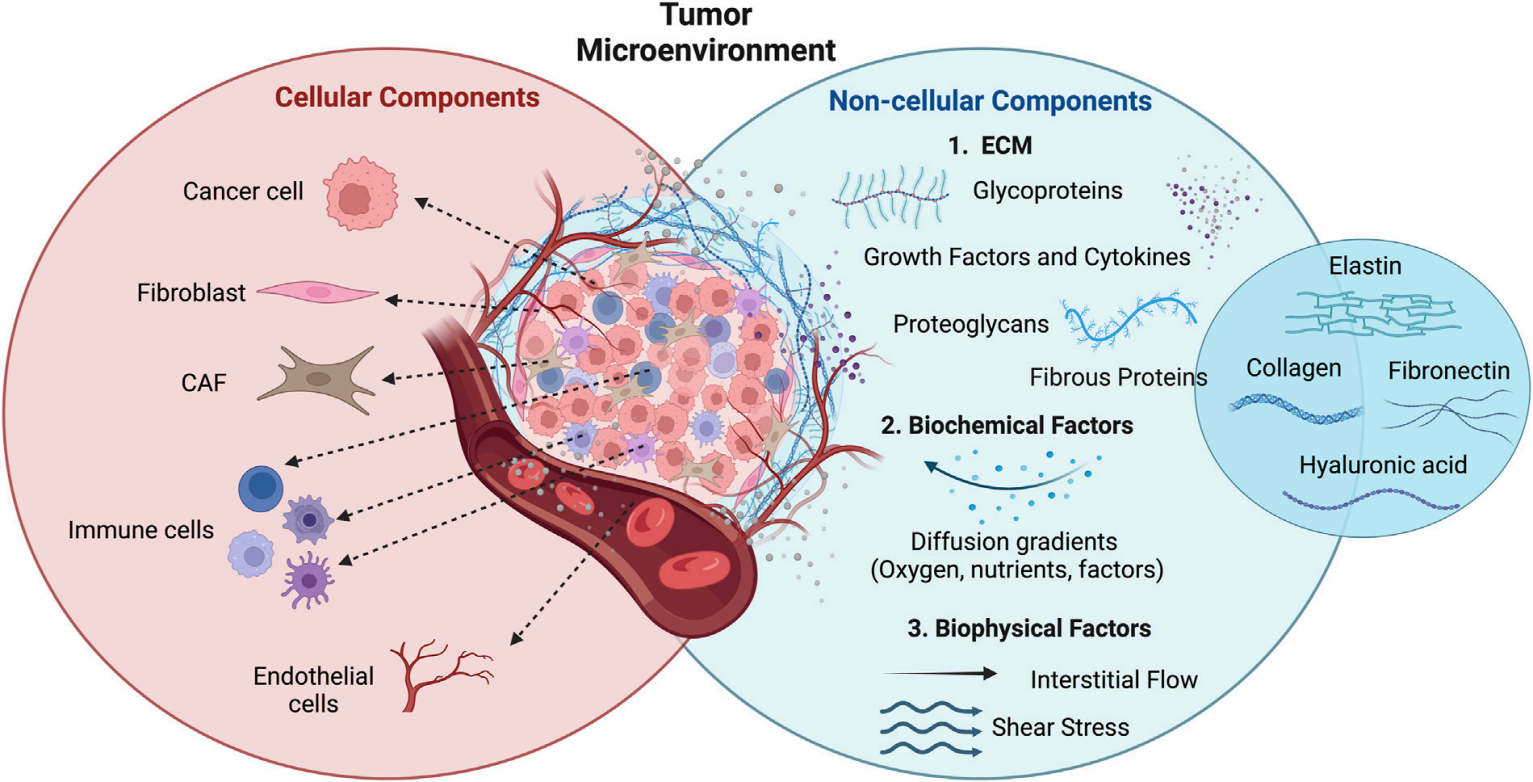
Representation of the TME components and their organization within the tumor mass. Cellular (cancer cells, fibroblasts, immune and endothelial cells, CAFs) and non-cellular (ECM, biochemical factors, and biophysical factors) components. Figure adapted from Benavente et al., 2020 in BioRender.com.

According to the World Health Organization (WHO), in 2022 there were nearly 20 million new cases of cancer worldwide and, it is expected that this number increase to 35 million in 2050 (Bray et al., 2024; World Health Organization, 2024). These data highlight the urgency in understanding cancer biology and its mechanisms of initiation and progression, to develop more effective biomarkers for early diagnostics and novel therapeutic systems (Bray et al., 2024). Cancer cells are surrounded by different components (cellular and non-cellular), making up the tumor microenvironment (TME) (Figure 2) (Roma-Rodrigues et al., 2019). Regarding the cellular components of TME, these include adipocytes, lymphocytes, endothelial cells from the blood and lymphatic vasculature, pericytes, cancer-associated fibroblasts (CAFs), and tumor-associated macrophages. Within the non-cellular part of TME, the main component is the extracellular matrix (ECM), which is a complex assembly of various macromolecules, like growth factors, fibrous proteins (collagen, elastin, and fibronectin), proteoglycans, glycoproteins and cytokines (Belli et al., 2018; Roma-Rodrigues et al., 2019; Baghy et al., 2023). The main functions of ECM include the support of the 3D structure, maintenance of tissue homeostasis, and modulation of cell-to-cell and cell-matrix interactions, thereby influencing cell differentiation, survival, and proliferation (Belli et al., 2018; Baghy et al., 2023). Besides the ECM, biochemical factors, such as diffusion gradients of molecules, signaling factors, oxygen and nutrients, and biophysical factors, like shear stress and interstitial flow, also play an important role in TME (Li et al., 2023; Manduca et al., 2023).

Overall, TME is responsible for the modulation of processes related to cell invasion and metastasis, tumor growth, immune responses, and mechanisms of drug resistance (Baghy et al., 2023). As mentioned, cancer is an intricate process that is dependent on the interaction between cancer cells and TME. Therefore, it is essential to explore new disease models capable of mimicking those interactions to provide more insights into the mechanisms of cancer progression and enhance the effectiveness of novel therapeutic screening (Burgos-Panadero et al., 2019; Babar et al., 2023).

## 2 Cell models

### 2.1 2D models

Over the past decades, new anticancer drug candidates have experienced significant attrition rates in clinical trials, largely due to their limited safety or/and lack of efficacy (Li et al., 2023). A contributing factor is the absence of disease models capable of accurately capturing the diversity and complexity of tumors. Preclinical studies, particularly those based on drug screening, heavily rely on 2D cell monolayers due to their ease of implementation, cost-effectiveness, reproducibility, versability, and compatibility with highthroughput screening (Manduca et al., 2023; Nayak et al., 2023; Tosca et al., 2023). However, as explained above and highlighted in Figure 1, these models do not fully recapitulate important features of the TME, including cell-to-cell and cell-matrix interactions, and cellular heterogeneity (Figure 2) (Jiang et al., 2023). Additionally, when cultured as monolayers, tumor cells receive nutrients and oxygen indiscriminately, which does not apply to an *in vivo* environment, leading to changes in gene and protein expressions, metabolism, and proliferation kinetics (Li et al., 2023; Sharma et al., 2023; Sharma et al., 2024). Conversely, animal models (being mice the most used) are more complex, displaying a complete physiological response. However, this response is murine-specific, and generally, these mice are often immunologically compromised, all of which may impair the predictiveness of the *in vivo* response in drug testing (Li et al., 2023). To address such concerns, a new generation of mice with human-like features has been recently developed (Li et al., 2021). Still, considerable research should be done to improve their recapitulative potential (Li et al., 2023; Sharma et al., 2023). Moreover, using animals for disease modeling and drug testing raises ethical issues, due to the growing awareness of animal suffering and sacrifice (Kiani et al., 2022). Recently, to circumvent some of the hurdles associated with these humanized mice, different groups (Al-Hamaly et al., 2023; Costa et al., 2024; Fieuws et al., 2024) have been using patient-derived xenograft (PDX) in zebrafish as models (Zebrafish Avatars) for drug screening and predicting individual treatment responses. By using zebrafish embryos, these models have several advantages, such as the speed of data acquisition, as tumor evolution and reaction to therapy can be evaluated within a timeframe that aligns with clinical decision-making and most importantly, revealing their anti-angiogenic and anti-metastatic potentials *in vivo* (Li et al., 2021; Al-Hamaly et al., 2023; Costa et al., 2024; Fieuws et al., 2024). Despite this enormous potential, standardization of methods and validation in larger clinical trials are needed before translation for personalized cancer medicine. While Zebrafish Avatars are making their way to the clinics, the establishment of other advanced and reliable models for accurate disease modeling, drug development/screening, and personalized medicine are also needed.

### 2.2 3D models

3D models are acknowledged as promising alternatives to conventional preclinical models. These advanced cell models exhibit increased recapitulative potential of the *in vivo* environment and are suitable for large-scale screening. These are considerably able to replicate numerous aspects found in native tumors, such as the 3D architecture of tissues, promoting the occurrence of cell-to-cell and cell-matrix interactions, cell polarization, formation of oxygen, nutrient and pH gradients, *de novo* ECM deposition and growth kinetics (Nayak et al., 2023; Brooks et al., 2024). Recent works reported that 3D models present similar gene expression patterns, drug resistance mechanisms, and signaling pathways activation status to those observed *in vivo* (Arutyunyan et al., 2023; Miller et al., 2023). These features demonstrate the potential that 3D cultures hold as improved disease models.

These advanced 3D models are generally categorized into spheroids or organoids, according to the native cell material from which the models originate. Spheroids are basic aggregates composed of cells, obtained from different sources, such as primary cells, tissue fragments, or established cell lines (Kim et al., 2023; Wang Y. et al., 2023). These cells aggregate, forming compacted and rounded structures with no specific function. Conversely, organoids derived from stem cells can create complex 3D clusters that self-organize and differentiate into tissue-specific cell types, often resulting in self-renewable tissue-like structures with similar organ functions (Fang et al., 2023). The subsequent sections offer a more in-depth explanation regarding the fundamental characteristics of these models, their main advantages, and challenges.

#### 2.2.1 3D spheroids

Spheroids are the most endorsed 3D models in tumor research due to their simplicity. These are microsized aggregates of closely-packed cells, that can originate from a diverse array of neoplasms (Manduca et al., 2023), namely, from brain (Wanigasekara et al., 2023; Heinrich et al., 2024), breast (Ahvaraki et al., 2024; Ascheid et al., 2024), cervix (Xu et al., 2023; Kumar et al., 2024), colon and rectum (Heydari et al., 2021; Valente et al., 2023), lung (Batista et al., 2024; Li et al., 2024), pancreas (Bano et al., 2024; Struth et al., 2024), and prostate cancer (Rakhmatullina et al., 2024; Song et al., 2024). Spheroids may be composed solely of tumor cells (homotypic spheroids), or also include more types of cells (heterotypic spheroids), such as fibroblasts, immune or endothelial cells (Manduca et al., 2023). Both cells, either from established cell lines or primary tumor tissue, can be used to develop 3D tumor spheroids. Generally, cell line-derived spheroids are easily handled and more adaptable for high-throughput screening. Conversely, primary tissue-derived spheroids are more troublesome to maintain, presenting limited lifespans and variable establishment rates (Tosca et al., 2023), but can be valuable tools to understand how genotypic variations can influence response to treatment, due to inter-patient variability. Additionally, these spheroids can also be applied to personalized medicine.

Spheroids can accurately mimic several *in vivo* features as already reported above, making them suitable models for more reliable cancer studies (Lee et al., 2023; Li et al., 2023). Strong cell-to-cell interactions and the presence of dense ECM hamper the transport of nutrients, oxygen, and other soluble molecules, leading to differential diffusion and exchange rates, particularly in spheroids larger than 400 μm (Li et al., 2023; Manduca et al., 2023). This results in the formation of different cell layers: a proliferating external region; a transitional sheet of quiescent cells; and a hypoxic, acidic, and necrotic core, as observed in small metastatic lesions and non-vascularized tumors (Valente et al., 2023; Li et al., 2023; Manduca et al., 2023). Such heterogenous multilayered organizations had a pivotal role in endorsing the application of spheroids as preclinical models in drug development and screening. The increased ECM deposition combined with the strong cell-to-cell interactions observed in these models acts as a physical barrier that hampers drug diffusion into spheroids (Tosca et al., 2023). Moreover, senescent cells in the inner zones of spheroids exhibit increased resistance to antiproliferative drugs compared to the proliferative cells at the periphery of the spheroids (Manduca et al., 2023).

3D tumor spheroids can be generated using scaffold-free or scaffold-based approaches (Li et al., 2023). Scaffold-free models enable cells to organize themselves and secrete ECM, mimicking natural interactions. Key scaffold-free techniques comprise agitation, hanging drop, liquid overlay, and magnetic levitation (Tosca et al., 2023). Scaffold-based systems use synthetic [e.g., polyethylene glycol (PEG), polycaprolactone (PLC), poly-L-lactic acid (PLLA) and poly(lactide-co-glycolide) (PLGA)] or natural polymers (e.g., collagen, alginate, Gelatin-Methacrylate (GelMA), laminin-rich ECM, hyaluronic acid and fibrin) (Rodrigues et al., 2018), to support cell growth and mimic the ECM, improving nutrient exchange for cancer cells (Manduca et al., 2023). While scaffold-based methods enable more complex spheroid production, scaffold-free models tend to be simpler and more adaptable to high-throughput screening (Tosca et al., 2023). Additionally, advances in microtechnology and microfabrication resulted in the creation of innovative techniques, such as microfluidics and bioprinting, enabling the production of controlled architectures (e.g., micro-vessel models) that mimic the interactions and the physiological environment of the cells (Rodrigues et al., 2018; Germain et al., 2022). Recently, Liu et al (2024) described the development of a microfluidic-based chip to generate breast cancer spheroids, assess treatment response, and measure cytokine secretion, demonstrating the potential of these new methods for high-throughput generation of spheroids and treatment studies (Liu et al., 2024). Furthermore, new types of biomaterials are also insurging as the next-generation of scaffolds for *in vitro* cancer modeling (Rodrigues et al., 2018). As an example, methacrylate-gellan gum (Me-GG) scaffolds are being produced through bioprinting technology for 3D models of lung cancer (Villata et al., 2023).

Although tumor spheroids offer a more precise depiction of tumor biology than traditional 2D models, they still face considerable challenges as preclinical tools. One major challenge is the absence of established procedures (like SOPs) for their generation and analysis, which leads to variability in size, shape, and cellular composition across different laboratories (Manduca et al., 2023; Tosca et al., 2023). Thus, impairing the interlaboratory comparison of results and consistent reproduction of the findings. Furthermore, replicating the complex TME, including factors like vascularization and immune cell infiltration, remains an ongoing challenge in spheroid models. Despite all the relevant TME features recapitulated by spheroids, several are still not addressed. These include the 3D architecture of the original tissue, tissue deformation, and interstitial fluid pressure, all of which can greatly impact cancer cell behavior (Manduca et al., 2023).

#### 2.2.2 3D organoids

The organoid formation process involves cell dissociation and re-aggregation in an *in vitro* 3D environment, forming clusters that can self-organize and differentiate in different cell types. Those clusters capture features of the *in vivo* microenvironment and are highly similar to actual organs (Corrò et al., 2020; Kim et al., 2020; Tang et al., 2022). Organoids may be derived from various sources, such as primary tissue biopsies, adult stem cells (ASCs, also denominated as primary tissue stem cells), embryonic stem cells (ESCs), and induced pluripotent stem cells (iPSCs). Compared with 2D and models, organoids enable increased patient specificity, being more easily handled and offering improved tumor behavior responses, which makes them advantageous for drug screening, personalized therapy, and diagnosis (Zhao Z. et al., 2022).

##### 2.2.2.1 Formation of organoids

Even though the origin of the organoids may be different, the formation process is similar for all of them. Overall, it consists of an initial tissue dissociation into single cells or clusters, followed by their embedment into a 3D matrix (like Matrigel^®^) and cultured in rich media supplemented with specific growth and differentiation factors (such as epidermal growth factor, fibroblast growth factors, R-spondin, Noggin and retinoic acid). Still, the specific formulation of cell media needs to be tailored to suit the unique requirements of each cell and organoid type being modelled (Drost and Clevers, 2018; Corrò et al., 2020; Kim et al., 2020; Tang et al., 2022). Tissue dissociation methods are categorized as enzymatic or mechanical methods (Jiang et al., 2023). The enzymatic methods are the most frequently used and require an enzymatic digestion to dissolve the ECM. The enzymatic cocktail composition may differ according to the tissue type, being enzymes like collagenase, elastase, and dispase are the most commonly applied. Although mechanical digestion is faster and more cost-effective, it often resultsin higher cell death and lower cellular recovery (Richter et al., 2021). Therefore, combining both methods can yield better results (Jiang et al., 2023).

To avoid direct contact with the dish and, consequently, the adherence of the cells to their surface organoids are formed in a suspension culture. To this end, various types of techniques can be employed, which are divided into scaffold-free and scaffold-based techniques, for spheroid formation (Luo et al., 2021; Górnicki et al., 2024). Scaffold-free methods involve creating droplets of a specific culture medium that contain cells, which hang from a plate due to gravity and surface tension. On the other hand, scaffolds are biological or synthetic hydrogels that can mimic ECM. For example, Matrigel^®^ is constituted by a jelly-like mixture of proteins and growth factors recovered from Engelbreth-Holm-Swarm (EHS) mouse sarcoma cells (Luo et al., 2021; Zhao Z. et al., 2022; Abuwatfa et al., 2024; Górnicki et al., 2024). Matrigel^®^ provides structure and enables cell communication, recapitulating the native features of tumors (Luo et al., 2021).

Cell culture medium is also an important factor for organoid development since its previous adaptation helps the cells to perform certain functions that 2D models are not able to recapitulate (Sato et al., 2009; Corrò et al., 2020). A study developed by Li et al. (1987) reported that breast epithelial organoids could form channels and cavities when grown on specific extract and were able to synthesize and secrete the milk protein (Li et al., 1987). This work has triggered interest and paved the way for many other works in organoids from different sources.

##### 2.2.2.2 Sources

**FIGURE 3:**
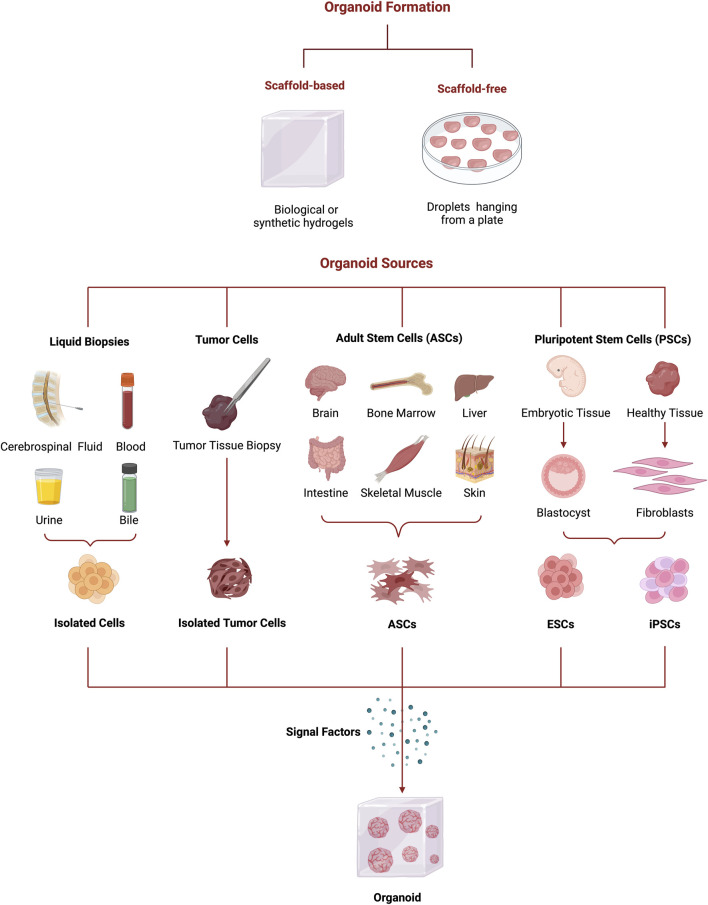
Schematic representation of organoids formation from several organoids’ sources (liquid biopsies, tumor biopsies, ASCs, and PSCs) to the different culture methods (scaffold-based and scaffold-free) and signal factors. Figure created with BioRender.com.

When forming organoids, the selection of the appropriate biological material is essential to guarantee the structure and functionality of the models. Therefore, the selection is dependent on the intended application (like disease modeling, developmental biology, drug screening, or personalized medicine). The different sources for organoids generation can be classified as conventional [e.g., pluripotent stem cells (PSCs), ASCs and primary tissue biopsies] and non-conventional (liquid biopsies) (Figure 3) (Yang et al., 2019).

Among all sources, PSCs are the most used. Examples include ESCs, which are retrieved from the inner cell mass of blastocysts, and iPSCs, obtained by reprogramming differentiated cells back into a pluripotent state (Narsinh et al., 2011; Lei and Schaffer, 2013; Takahashi and Yamanaka, 2013). The main feature of PSCs, that makes them highly valuable for organoid formation, is their pluripotency, allowing the creation of different organoid models, from brain to liver to intestinal organoids, each accurately reflecting the complexity and cellular diversity of the respective tissues (Paşca et al., 2015; Kim et al., 2020; Shankaran et al., 2021; Ouchi and Koike, 2023). Considering this ability, organoids derived from PSCs are extensively used to model diseases, since they accurately recapitulate the pathophysiology of conditions, such as neurodegenerative diseases, liver disorders, and gastrointestinal diseases in a controlled 3D environment (Jensen et al., 2009; Spence et al., 2011; Desbordes and Studer, 2013; Schwartz et al., 2016; Amin and Paşca, 2018; Silva and Haggarty, 2020). Still, applying PSCs to organoid generation comes with several challenges, the reprograming process being the major one. Achieving specific cell types with high efficiency and reproducibility is an onerous procedure, requiring precise and tight control over the differentiation environment since it can introduce mutations and alter gene expression patterns (Takahashi and Yamanaka, 2016; Shao et al., 2017). The risk of tumorigenicity, particularly with the use of oncogenic factors like c-MYC, also poses a significant hurdle (Okita et al., 2007; Yamanaka, 2020). Furthermore, the use of ESCs raises significant ethical concerns, and iPSCs provide a more ethically acceptable alternative (Moradi et al., 2019; Rodriguez-Polo and Behr, 2022).

While PSCs are commonly used for organoid generation due to their high abundance and ability to differentiate into any cell type, ASCs are multipotent, meaning they can only differentiate into a restricted variety of cell types associated with their tissue of origin (Young and Black Jr, 2004; Sobhani et al., 2017). Despite that, they require less cellular transformation processes, allowing to generate organoids that better capture the configuration, function and cellular composition of the native tissue (Clevers, 2016; Brassard and Lutolf, 2019; Wu et al., 2023). ASCs-derived organoids have a great impact in several applications such as disease modeling, particularly in tissue-specific diseases, drug testing, and regenerative medicine, considering that they provide a high degree of tissue specificity. In addition, the use of ASCs also reduces ethical concerns associated with embryonic sources and can be patient-specific, allowing personalized medicine approaches (Jin et al., 2024).

Nevertheless, primary tissues are the best source for organoids formation with higher resemblance to the native tissue, since they allow cells to preserve their original genetic, epigenetic, and phenotypic characteristics (Huang et al., 2015; Mo and Izpisua Belmonte, 2019). The process of obtaining primary tissues typically involves invasive procedures such as needle and endoscopic biopsies or surgical resections, which afterward are dissociated and cultured as previously explained (Gao et al., 2018; Nuciforo et al., 2018; Seidlitz et al., 2019). Organoids derived from primary tissue can be composed of a mixture of epithelial, immune, stromal, and other specialized cells, depending on the tissues’ origin, leading to intrinsic heterogeneity. This feature has become relevant since it allows the development of patient-specific models to evaluate individual disease progression and develop tailored treatments accounting for the genetic and phenotypic landscape of each patient (Ooft et al., 2019; Hao et al., 2022; Zu et al., 2023; Smabers et al., 2024). Nevertheless, the implementation of organoids as pre-clinical models has been hampered by the finite proliferative capacity of primary cells, preventing expansion and long-term culture; the necessity for invasive procedures to obtain tissue samples; limited representation of rare cell types; poor functional differentiation; low phenotypic stability; and poor uniformity and consistency across organoid replicates (Jiang et al., 2017; Hu et al., 2018; Kasagi et al., 2018; Calà et al., 2023).

To surpass the hurdles associated with the obtention processes of cell samples, novel minimally invasive sources of biological material have been explored, particularly liquid biopsies (Junqueira-Neto et al., 2019; Alix-Panabieres, 2020). Liquid biopsies can be acquired through blood, with the isolation of circulating tumor cells (CTCs), or other body fluids (e.g., bile, cerebrospinal fluid, and urine) (De Mattos-Arruda et al., 2015; Reckamp et al., 2016; Alix-Panabières and Pantel, 2021; Dell’Olio et al., 2021). CTCs isolation requires highly specialized and sensitive techniques, due to the scare amount of target cells in these fluids (Cristofanilli et al., 2005; Chinen et al., 2013; Harb et al., 2013; Harouaka et al., 2013; Abdulla et al., 2018; Neves et al., 2019). Bile, endoscopic or percutaneous procedures can collect samples containing epithelial cells from the biliary tract (Saxena et al., 2015; Zhao X. et al., 2015). Cerebrospinal fluid is obtained via lumbar puncture, and urine samples are collected non-invasively (Pearce, 1994; Dörrenhaus et al., 2000; Kaeffer, 2011; Engelborghs et al., 2017). Organoids originating from those sources are extremely valuable as pre-clinical models (Drost et al., 2015; Broutier et al., 2017; Zhao et al., 2017). For instance, CTCs from blood are used as a model to the study of metastatic cancers (Bardelli and Pantel, 2017; Yang et al., 2019), bile cells allow the study of cholangiocarcinoma and cholangiopathies (Soroka et al., 2019b; Soroka et al., 2019a; Kinoshita et al., 2023), urine cells offer insights into kidney diseases and bladder cancer (Walz et al., 2023), and cerebrospinal fluid cells can be used to model brain barrier permeability and neuroepithelial barrier and secretory functions (Pellegrini et al., 2020). Despite the potential highlighted by these organoid models, the lack of standardized protocols for using liquid biopsies in organoid formation, as variations in collection, processing, and storage of samples can lead to inconsistent results, creating difficulties in reproducibility and comparison between studies (Vasseur et al., 2021; Lawrence et al., 2023; Sidaway, 2023).

In the future, novel techniques and improvements of the already established methods may expand applications of organoids as alternative cancer models. These may include advances in tissue engineering and biomaterials, progress in the microfluidic devices (Qiao et al., 2024), improvement of the methods to acquire the biological materials (especially in the cases of ASCs, primary tissues, and liquid biopsies) (Martins et al., 2021; Bex and Mathon, 2022; Shegekar et al., 2023), and standardization of procedures for organoid integration in drug screening platforms and CRISPR-based gene editing (Matano et al., 2015; Sun et al., 2019; Takeda et al., 2019; Truong et al., 2019).

##### 2.2.2.3 Types of organoids

The types of organoids can be differentiated by their tissue origin, which can include various organs, including the gastrointestinal (GI) tract, stomach, pancreas, intestine, liver, brain, retina, kidney, and others (Figure 3) (Ashok et al., 2020). It is crucial to comprehend the microenvironment in which the initial cellular population is placed to produce viable organoids (Zhao Z. et al., 2022). GI tract organoids have additional complexity since they require a deeper understanding of homeostasis and tube’ segment development, which are formed during human development, thus involving different molecular mechanisms and active signaling pathways (Günther et al., 2022).

Intestine organoids derived from ASCs have an established long-term culture protocol, in which WNT and EGF play a key role in their maintenance and BMP promotes villi differentiation. In contrast, PSCs-derived organoids require a new protocol to support their growth and maturation (Merker et al., 2016; Zhang et al., 2021). Studies involving the grafting of gut organoids into mice demonstrated sustained integration, underscoring their tissue-repairing capabilities (Drost and Clevers, 2018). Given the molecular and physiological similarities of both stomach and intestinal, the protocols for their formation are almost identical (Merker et al., 2016). For both liver and pancreatic organoids, it has been shown that they can be generated through cycling LGR5+ cells in Matrigel^®^ while supplemented with the appropriate cell medium (Huch et al., 2013; Hindley et al., 2016).

Brain organoids are the most difficult to produce due to the complex system composed mainly of neurons and glial cells (Tang et al., 2022). Consequently, these models are still not well established, and, for this reason, most brain studies still rely on 2D cultures or simple aggregates (Corrò et al., 2020). Recently, Lancaster et al. (2013) were able to originate “mini-brains” in Matrigel^®^, by supplementing the medium with Hedgehog, FGF, BMP, and WNT growth factors (Lancaster et al., 2013).

##### 2.2.2.4 Advantages and limitations

**FIGURE 4:**
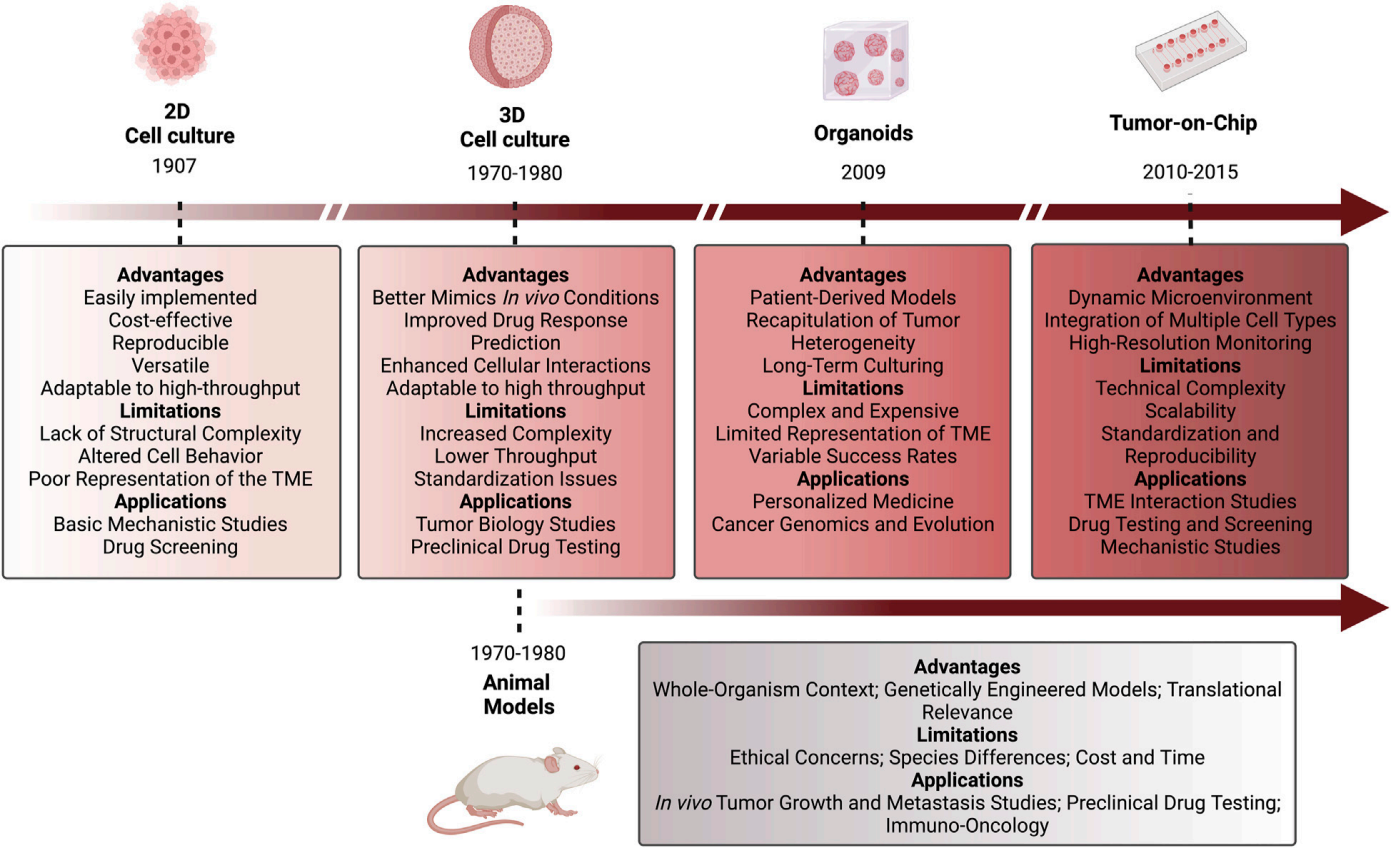
Chronological representation of the evolution of 2D and 3D cellular models and animal models. Description of the advantages, limitations, and applications of each represented model. 2D cell culture models were the first to be implemented in research (1907), followed by 3D cell cultures and animal models (1970–1980), then organoids (2009), and lastly Tumor-on-Chip models (2010–2015). Figure created with BioRender.com.

Traditional models of human development and diseases typically involve 2D cell and tissue transplantation into *in vivo* models, including genetically engineered mice and PDXs. However, those alternative models do not completely recapitulate the human conditions (like tissue architecture and complexity), and some are too expensive and take too long to be produced. PDOs emerge as a viable alternative to those models (Huang et al., 2021; Taelman et al., 2022). Once the culture conditions have been optimized taking into consideration the origin of the tissue, PDOs can be easily generated by collecting samples through needle biopsies, urine, or bronchial wash material (Kaushik et al., 2018; Corrò et al., 2020; Jensen and Little, 2023). Compared to the 2D cultures, organoids can recapitulate the 3D structure, maintaining the heterogeneity and cell function, and do not induce significant genetic changes. Organoids are also less expensive and less time-consuming than PDXs and genetically engineered mouse models, therefore being more suitable for high-throughput screening (Figure 4) (Heydari et al., 2021; Huang et al., 2021).

Regarding their limitations, organoids still fail to completely mimic all TME components and interactions, which compromises the observed outcome when, for instance, evaluating the response of a tumor to immunotherapy (Table 1; Figure 4). The different proliferation rate of each cell type and the requirement for specific growth factors and oxygen also prevents the development of more sophisticated models (Kaushik et al., 2018; Huang et al., 2021). Also, the lack of vascularization is another limitation of organoids, confining the maximum size attained (Ashok et al., 2020). Nevertheless, recent studies opened a window to overcome this organoid’s limitation. For example, Neal et al. (2018) used a gas-fluid-surface method to culture organoids above a layer of fibroblasts, prolonging the survival time of fibroblasts and immune cells (Neal et al., 2018). An alternative study focused on co-cultures of organoids and peripheral blood lymphocytes to measure the T cell-mediated killing proficiency of matched tumor organoids (Dijkstra et al., 2018).

**TABLE 1:** Brief description and main advantages and limitations of the most used cancer models.

Model	Description	Advantages	Limitations
2D-cell monolayer	Cells from establish cell-lines or primary cells are cultured in a flat, 2D monolayer on plastic or glass surfaces	Simple, cost-effective, easy to handle, well-established, allows for high-throughput screening	Lacks the complexity of the 3D tissue architecture Limited cell-cell and cell-matrix interactions, reducing physiological relevance Lacks diffusion gradients and hypoxia Static model
3D Tumor spheroids	Cells from one or multiple cell-lines are cultured in 3D aggregates that mimic the structural organization of solid tumors	Better recapitulation of the 3D architecture and cell-cell interactions Presence of diffusion gradients of oxygen (hypoxia), nutrients and signal molecules Closer to physiological tumor behaviour than 2D cultures	Limited complexity compared to *in vivo* tumors More difficult to handle and less control over replicates (size and shape) Limited ability to incorporate stromal and immune components Static model Not all cells can form aggregates
Organoids	3D cultures derived from primary tissue biopsies, ASCs, ESCs, and iPSCs, retaining much of the architecture, genetic makeup, and heterogeneity of the original tumor	High physiological relevance, mimicking patient-specific tumor characteristics Useful for personalized medicine and drug screening Preserves key features of tumor heterogeneity	Technically complex, cumbersome and time-consuming to establish Lacks standardized protocols for organoid formation and maintenance High variability between organoid cultures Lack of immune system components unless co-cultured Static model
Tumor-on-Chip	Microfluidic devices that recreate a miniaturized TME by combining 3D cell cultures with fluid flow and mechanical forces	Mimics both the 3D structure and physiological conditions, such as fluid flow and shear stress Allows for dynamic study of drug transport, cell interactions, and mechanical cues Can integrate multiple cell types and ECM components, creating a more realistic tumor model	Technically challenging and requires specialized equipment and trained personel High costs for materials and equipment Lack of standardized protocols for platform development and operation Limited scalability for high-throughput applications
Animal models	*In vivo* models where human tumors are implanted or induced in animals (commonly mice), or genetically engineered models that spontaneously develop cancer	Provide a full biological context, including immune system interactions and systemic factors Allows for studying metastasis, tumor progression, and immune responses Essential for preclinical validation of therapeutics	Expensive, time-consuming, and subject to ethical concerns Differences between animal and human biology may reduce translational accuracy Limited ability to perform high-throughput screening

Disadvantages in organoid systems, such as the use of Matrigel^®^, mice-specificity of some tumors, batch-to-batch variability, high cost, and safety issues, may complicate the development of standardized protocols, as the ones established for the 2D cultures (Kozlowski et al., 2021), which have been hampering broader application of organoids in drug development and regenerative medicine (Kim et al., 2022). Additionally, the culture medium requires a cocktail of different growth factors, which may need several optimizations, to prevent the disruption of the natural morphogen gradients of the tissues (Ashok et al., 2020; Huang et al., 2021). Therefore, the use of alternative scaffolds with well-defined composition has been investigated, such as natural matrices that would not have the batch-to-batch variability that Matrigel^®^ has or the utilization of synthetic hydrogels, since these enable the manipulation of biochemical and biophysical matrix properties (Kozlowski et al., 2021 and for review see Ashok et al., 2020). Finally, the necessity to fine-tune several parameters contributes to the elevated cost of organoid development as well as the complexity of their scale-up (Ashok et al., 2020).

Overall, even though organoid technology is still in its early stages of maturation when compared to other models, its potential in pharmaceutical drug testing and molecular medicine is very promising.

## 3 Main applications of each model in cancer research

The various models described can be applied in several applications. Depending on the purpose of the study, different models may be required. The tendency is the use of more complete and complex models (e.g., PDOs and Tumor-on-Chip) to achieve the most accurate response possible. The following sections will focus on the main applications of each model in drug development and screening and personalized medicine, as well as the principal advantages and challenges that have been found over the years.

### 3.1 Drug development and screening

As already stated, one of the major challenges preventing the development of efficient anticancer drugs is the poor translatability of preclinical results, typically obtained from 2D cell cultures, xenografts, and animal models. Consequently, advanced 3D cell models, such as tumor spheroids and organoids, have been endorsed as alternative models to develop preclinical studies (Hirschhaeuser et al., 2010; Hamilton and Rath, 2019; Driehuis et al., 2020a; Schueler et al., 2022).

Despite their advantages in biomedical applications, the use of spheroids and organoids is still barely reported (Schueler et al., 2022). Challenges remain since these 3D models have yet to be routinely included in the drug screening procedures, and the animal models were not entirely replaced by them (Heinrich et al., 2021). The ability of tumor spheroids and PDOs to recognize promising drug candidates that failed in traditional 2D cell assays, before *in vivo* experiments has been widely highlighted over the last years (Hirschhaeuser et al., 2010; Pauli et al., 2017; Praharaj et al., 2018; Driehuis et al., 2019; Jacob et al., 2020; Qu et al., 2021; Qu et al., 2024; Chen et al., 2022; LeSavage et al., 2022; Cabeza-Segura et al., 2023; Wang H.-M. et al., 2023).

Lately, the most promising application of 3D organoids in cancer therapy involves drug screening of novel agents and personalized medicine. Established organoid repositories can be leveraged for screening assays, hastening the discovery of potentially effective agents from new compounds or finding alternative applications for existing drugs. Moreover, organoids improve the study of combinatory drugs and new methods to overcome drug resistance (Tosca et al., 2023).

Currently, the standard models for anticancer drug development and screening (e.g., small molecule drugs and chemotherapy agents) are 2D cancer cell lines and tumor tissue animal transplantation (e.g., xenografts) models (Kitaeva et al., 2020; Langhans, 2021; Zhang et al., 2023; Qu et al., 2024). Xenograft models are useful in drug screening since they retain tumor heterogeneity and simulate the TME. Still, they present major disadvantages like long experimental periods, high costs, and unsuitability for high-throughput screening (Gao et al., 2015; Langhans, 2021; Zhao H. et al., 2022). Traditional toxicological screening methods using 2D cultures and *in vivo* models often fail to predict human adverse reactions precisely. Being organ toxicity a major reason for drug failure and withdrawal, even post-approval (Raghavan et al., 2021). Hence, organoids have become invaluable in drug research and development, improving drug toxicity detection, high-throughput screening, and pharmacokinetic studies (Clevers, 2016; Boretto et al., 2019; Saito et al., 2019; Driehuis et al., 2020b; Brooks et al., 2021; Ren et al., 2022; Yuan et al., 2022; Yang and Yu, 2023).

Advances in *in vitro* tumor models, including 3D spheroids and organoids, have significantly hastened the drug discovery and development process (Qu et al., 2024). Some studies have shown that spheroids better recapitulate drug responses in xenograft mice compared to 2D cell cultures. However, these studies only conducted qualitative comparisons with a limited number of cell lines and drugs. A more comprehensive analysis incorporating a wider variety of cancer cell lines and anticancer agents is necessary (Broutier et al., 2017; Qu et al., 2024).

Regarding organoid models, several studies have comparatively assessed drug responses obtained in both PDOs and PDXs, with the original patients’ responses obtained in the clinical scenario, showing good consistency across models. Still, the small sample sizes, different experimental designs (e.g., drug concentrations and exposure time), variation in the protocols for PDO culture, and varying efficacy thresholds used in PDOs, PDXs, and in patients may attenuate these findings. Thus, more wide-ranging studies with quantitative data and previously established criteria are needed to accurately assess the value of PDOs as pre-clinical models (Kita et al., 2019; Qu et al., 2024).

Nevertheless, both spheroids and organoids have the potential to become standard cancer models, providing clinical data of increased relevance, and enhancing the translational effectiveness of preclinical studies. Ultimately, widening treatment options for cancer patients, while reducing the exploit of animal models (Tosca et al., 2023).

### 3.2 Personalized medicine

As already mentioned, personalized medicine is another promising application of 3D cultures. In the past years, significant effort has been made to develop accurate cancer models. Such was achieved by transitioning simplistic monolayer cultures to sophisticated 3D systems, mostly relying on primary tumor sources, like PDOs. Hopefully, continued improvements in tumor modeling could lead to the development of reliable 3D “patient avatars,” that might help in guiding therapeutic selection for cancer patients (Fong et al., 2017). In this regard, Pauli et al. (2017) established a precision cancer care platform, by combining whole-exome sequencing with a living biobank for high-throughput drug screenings using PDOs. Conversely, Ho et al. (2018) highlighted advancements in 3D organoid technology, showing its use in cancer and other disorders, like modeling complex hereditary diseases.

Organoids also play a significant role in the discovery of novel tumor biomarkers (Huang et al., 2020; Ukai et al., 2020; Low et al., 2021). A meta-analysis conducted by G. E Wensink in 2018, analyzed 17 studies of PDOs for testing personalized tumor response (Beutel et al., 2021). Results from those studies revealed that PDOs hold tremendous clinical value as a predictive tool for cancer patients’ personalized treatment response (Qu et al., 2024). As such, integrating these models with individual genomics data and drug development pipeline could expedite personalized medicine findings, rendering for tailored treatment regimens, with improved efficacy and reduced side effects, ultimately enhancing patient survival and quality of life (Qu et al., 2024). Supplementary Table S1 includes examples of the main applications of 3D models for cancer therapy (Tosca et al., 2023).

Although it is improbable that these 3D cancer models will fully replace animal models at this time, they are anticipated to become a crucial intermediary between 2D *in vitro* and *in vivo* models. Such has been endorsed in the latest FDA Modernization Act 2.0, which admits the use of alternative models like spheroids and organoids in place of certain animal studies, could increase their popularity and standardization [Booij et al., 2022; S.5002—117th Congress (2021–2022): FDA Modernization Act 2.0., 2022]. Altogether, using 3D models to eliminate ineffective treatments earlier could reduce the costs associated with cancer research, animal use, and ethical concerns (Qu et al., 2024).

### 3.3 Gene modulation and editing

PDOs with monogenic diseases can be edited to correct the disease-causing mutations. Such was proved by Schwank, G., et al (2013) while using CRISPR/Cas9 to correct a faulty cystic fibrosis transmembrane conductance regulator gene in intestinal organoids from two cystic fibrosis patients (Schwank et al., 2013; El Harane et al., 2023). Live-cell microscopy showed that the corrected organoids expanded rapidly, unlike the uncorrected controls (Schwank et al., 2013).

CRISPR/Cas9 has proven nifty for activating or deactivating tumor suppressor genes, inactivating oncogenes, and correcting disease-causing mutations. The use of this technique has significantly improved the outcomes of creating new organoid and cell line models. However, despite its promise, phenotypic rescues using CRISPR/Cas9 in human organoids remain limited. Developing transplantation techniques for genome-edited PDOs is essential for clinical applications (El Harane et al., 2023).

### 3.4 Immunotherapy

Dysregulations in the TME contribute to frequent relapse when conventional cancer treatments are applied. Forecasting when patients will not respond to certain therapies is essential for enhancing cancer therapy (El Harane et al., 2023). In the last years, immunotherapy has demonstrated encouraging outcomes in various cancers, such as melanoma and lung cancer. However, its effectiveness varies among patient groups due to the complex nature of the TME, which can lead to resistance to therapeutic agents (Kirkwood et al., 2012).

Utilizing 3D models for evaluating immunotherapies offers a promising alternative strategy, since CAR-T therapy has shown encouraging results in certain liquid tumors but remains less effective in solid tumors (Ma et al., 2019). As so, reliable preclinical models are needed to replicate human cell surface markers and evaluate CAR-T efficacy on these new targets (Ning et al., 2024). The co-culture model of tumor organoids and CAR-T cells offers advantages over traditional preclinical models and has been used as a supplementary approach in research. Still, the current literature indicates that this model has limitations. Recently, Mei et. al (2024) listed clinical trials involving the application of PDOs for immunotherapy registered in EudraCT and in Clinicaltrials.gov (Mei et al., 2024). For example, a system named BEHAV3D was created to investigate the interactions between immune cells and PDOs through imaging and transcriptomics (Dekkers et al., 2023; El Harane et al., 2023). BEHAV3D showed the ability to trace over 150,000 engineered T cells with PDOs and researchers used it to examine cancer metabolome-sensing TEG cells (αβ T cells engineered with a γδ TCR). The results have shown significant variation in TEG cell killing effectiveness across PDO cultures from biobanks. They also found that the molecular mechanisms and response of cellular immunotherapy varied among different PDO cultures and even among individual organoids within the same culture. This highlights the platform’s ability to capture both inter- and intra-patient heterogeneity, a major challenge in treating solid tumors. Additionally, they showed that type I INF can prime resistant organoids for TEG-mediated killing (El Harane et al., 2023).

Despite the limitations described above, organoids have been used in several completed and ongoing clinical trials. These trials aimed at establishing or improving protocols for organoid generation, developing biobanks, extending the understanding of disease-specific patterns, seeking new potential therapies, and pursuing novel personalized treatments (Supplementary Table S2) (Foo et al., 2022). All these clinical trials highlight the value of organoids as a new “golden standard” for cancer modeling. As research continues, the integration of organoids into clinical trials is likely to expand, potentially leading to more effective and tailored therapies.

### 3.5 Tumor-on-chip (ToC)

The leveraging of microfluidics and tissue engineering allowed us to achieve an important milestone in cancer modeling, with the development of Tumor-on-Chip (ToC) technology (Giannitelli et al., 2024). ToC is a promising candidate to replace conventional experimental models, providing a more comprehensive and realistic model by integrating physical, chemical, and biological cues of tumor development and metastasis (Dsouza et al., 2022; Liu et al., 2022). The implementation of ToC can bridge the gap between traditional cell cultures and *in vivo* animal models, offering unprecedented potential in the realms of drug discovery, cancer research, and personalized medicine (Lovitt et al., 2014; Riedl et al., 2017; Chaicharoenaudomrung et al., 2019; Schuster et al., 2020; Vulto and Joore, 2021).

The milestones leading to the development of ToC devices comprise advancements in microfabrication and microfluidics. ToC integrates microfluidics, biomaterials, and living cells to create micro-engineered environments that closely mimic the *in vivo* TME (Table 2) (Liu et al., 2021). These devices can simulate various physiological conditions, such as nutrient and oxygen gradients (Chen et al., 2011; Brennan et al., 2014), mechanical forces (Lanz et al., 2017), and cellular interactions (Menon et al., 2014; Zou et al., 2015; Manoharan et al., 2024), providing a more accurate platform to enhance our understanding of cancer biology, reduce the time and cost associated with drug development, and improve clinical outcomes by enabling more effective and personalized treatment strategies (Lovitt et al., 2014; Vulto and Joore, 2021).

**TABLE 2:** Key components of ToC devices and their respective functionality.

Key components	Functionality
Microfluidic system	Enables precise control over fluids at the microscale. They facilitate the recreation of complex biological environments by controlling factors like nutrient gradients, shear stress, and oxygen levels
Cell culture	ToC systems support 3D cell growth with different cell types, which better replicates the architecture and function of real tumors. This includes proper cell-cell and cell-matrix interactions, as well as the development of environmental niches
Materials	Common materials include poly(dimethylsiloxane) (PDMS), Polymethyl methacrylate (PMMA), Polycarbonate (PC), Hydrogels (e.g., collagen, gelatin, alginate, Matrigel^®^), glass and Paper-based materials due to their biocompatibility and ease of use, although newer materials like polystyrene (PS) are being explored for their superior properties

The first microfluidic chip was developed in the 1980s, and the subsequent integration of living cells into these devices in the early 2000s (Whitesides, 2006). More recently, multi-organ devices are being developed by incorporating patient-derived cells and tissues, enhancing the relevance of these models for targeted medicine (Xu et al., 2016; Giannitelli et al., 2024). This combination of technologies endorsed the development of intricate platforms that replicate the dynamic environment of tissues and tumors, improving the reliability of preclinical studies (Ronaldson-Bouchard and Vunjak-Novakovic, 2018; Turetta et al., 2018).

Typically, ToC devices are fabricated using advanced microfabrication techniques, including photolithography (Xu et al., 2016), soft lithography (Xia and Whitesides, 1998; Silverio and Cardoso de Freitas, 2018), 3D printing (Lee and Cho, 2016; Yang et al., 2017; Steinberg et al., 2023) and laser ablation (Hsieh et al., 2017; Mansour et al., 2023). Each fabrication method offers unique advantages and drawbacks, making them suitable for different aspects of ToC device creation (Table 3). The choice of the technique depends on the specific requirements of the study, including precision, material compatibility, cost, and scalability. Integrating multiple techniques can often provide a balanced approach, leveraging the strengths of each to overcome individual limitations.

**TABLE 3:** Overview of the principal fabrication methods used to develop ToC devices.

Method	Description	Advantages	Limitations
Photolithography	Process used to transfer geometric patterns onto a substrate using light. It entails applying a photosensitive material known as photoresist to the substrate, exposing it to light through a mask, and developing the pattern by removing either the exposed or unexposed regions of the photoresist	High Precision and Resolution: Capable of producing extremely detailed and accurate microstructures, essential for replicating complex biological environments Scalability: Suitable for mass production, allowing for the creation of numerous identical chips Compatibility with Various Materials: Applicable to a wide variety of materials, including polymers, silicon and glass enabling diverse applications	Cost: High initial setup costs due to the need for cleanrooms and specialized equipment Material Limitations: Not suitable for all biocompatible materials, particularly some soft hydrogels Complexity: Requires multiple steps and precise alignment, increasing the complexity and potential for errors
Soft lithography	Soft lithography uses a flexible elastomeric stamp (usually made of PDMS) to transfer patterns onto a substrate. The stamp is created by casting PDMS on a master mold, which contains the desired microstructure	Flexibility: Can produce a variety of microstructures using soft, elastomeric materials like PDMS, which are biocompatible and transparent Cost-Effective: Less expensive than photolithography, as it does not require cleanroom facilities for every step Ease of Use: Simplified process that allows for rapid prototyping and modifications	Resolution Limitations: Lower resolution compared to photolithography, which may limit the precision of microstructures Deformation: Elastomeric materials can deform under pressure, potentially altering the microenvironment Reproducibility: Variability in the fabrication process can lead to inconsistencies between chips
3D printing	3D printing creates objects layer by layer from a digital model. There are several methods available, such as stereolithography (SLA), fused deposition modeling (FDM), and digital light processing (DLP)	Customization: Allows for highly customizable and complex designs tailored to specific research needs Material Variety: Can use various biocompatible materials, including hydrogels and biodegradable polymers Integration: Enables the incorporation of multiple functionalities within a single device	Resolution: Generally lower resolution compared to lithography, though this is improving with advances in technology Mechanical Properties: Printed structures may lack the mechanical strength and stability required for some applications Speed: Some 3D printing processes can be time-consuming, particularly for high-resolution or large-volume production
Laser ablation	Laser ablation uses a focused laser beam to remove material from a substrate by vaporization or sublimation. It is a precise method for cutting or engraving microstructures	Precision: High precision in cutting and structuring materials, useful for detailed microstructures Versatility: Applicable to a wide range of materials, including metals, polymers, and ceramics Minimal Contact: Non-contact process reduces contamination risk and preserves material integrity	Thermal Damage: Potential for thermal damage to materials, which can affect biocompatibility Cost: Requires expensive equipment and maintenance Scalability: Less suitable for mass production compared to lithographic methods

Materials commonly used include PDMS (Ng et al., 2002), hydrogels (Hoch et al., 2013), and other biocompatible polymers (Ren et al., 2013; Terrell et al., 2020). In the same manner as the fabrication methods, each material has its advantages and limitations, affecting their suitability for different applications. Generally, PDMS is the most popular material for its flexibility and ease of fabrication, but its chemical nature leads to the absorption of small hydrophobic molecules, narrowing its application in drug studies (McDonald and Whitesides, 2002; Auner et al., 2019). Conversely, PMMA and PC provide better mechanical strength but lack gas permeability, limiting the oxygen supply to the cells (Altmann et al., 2011; Palacio-Castañeda et al., 2020; Rodriguez et al., 2020; Bērziņa et al., 2021). Hydrogels offer a realistic 3D environment but have stability and reproducibility issues (Song et al., 2014; Chen et al., 2020; Terrell et al., 2020). Glass provides excellent optical clarity but is brittle (Aralekallu et al., 2023), while paper-based materials are low-cost and easy to handle but have poor optical properties for imaging, are less durable and offer less control over fluid flow (Ren et al., 2013; Ren et al., 2014). More recently, PS has also been explored due to its superior properties (Berthier et al., 2012). Among them, the ease of fabrication with more consistent and predictable surface properties than PDMS and PMMA, while allowing effortless molding and surface-treatment to enhance cell adhesion or create specific patterns for cell growth. The excellent optical transparency, similar to glass, makes it suitable for high-resolution imaging and microscopy. Additionally, PS also shows high chemical resistance, comparable to PMMA and PC, outperforming PDMS, which can absorb small hydrophobic molecules. Overall, the choice of material often depends on the specific requirements of the experiment and the desired balance between ease of use, biocompatibility, and mechanical properties (Ren et al., 2013).

These materials and techniques can be incorporated into several designs to replicate the architecture and functionality of tumors (Ronaldson-Bouchard and Vunjak-Novakovic, 2018). The design and functionality of the devices vary with the desired application and tumor models in study but generally include features such as microchannels, wells, and chambers to culture cells and tissues (Whitesides, 2006). Additionally, devices with increased complexity can be developed by integrating sensors, controllers, and imaging modules (Table 4) (Ronaldson-Bouchard and Vunjak-Novakovic, 2018). Altogether, this allows for precise control over the architecture and functionality of the chips.

**TABLE 4:** Description, function, and used materials of components of ToC devices that can be incorporated into a whole system.

Device part	Description	Function	Material/Type
Microfluidic Channels	Network of small channels through which fluids, cells, and nutrients flow	Simulate blood flow, nutrient and oxygen transport within the TME and control shear force	Typically made from PDMS, glass, or other biocompatible polymers
Cell Culture Chambers	Specific regions where tumor cells are cultured	House the tumor cells and recreate the 3D architecture of tumor tissues	PDMS, hydrogels (collagen, Matrigel^®^), or other matrix materials to support 3D cell growth
Biomimetic Extracellular Matrix	A scaffold that mimics the natural ECM of tissues	Provide structural support and biochemical signals to cells, enabling 3D cell culture	Collagen, gelatin, alginate, Matrigel^®^, and synthetic hydrogels
Substrate or Base Material	The foundational layer on which the entire device is built	Provide structural integrity and support for the microfluidic and cell culture components	Glass, silicon, PDMS, and other rigid or semi-rigid materials
Microenvironment Control Systems	Systems to control various environmental factors such as oxygen levels, pH, and temperature	Closely mimic the physiological conditions of the TME.	Microfluidic pumps, valves, sensors, and heating elements
Fluidic Pumps and Valves	Devices to control the flow of fluids within the chip	Deliver nutrients, remove waste, and introduce drugs or other substances in a controlled manner	Peristaltic pumps, syringe pumps, and on-chip micropumps
Sensors and Detectors	Embedded sensors to monitor various parameters such as pressure, oxygen concentration, and pH	Provide real-time feedback and ensure optimal conditions for cell growth and experimentation	Optical sensors, electrochemical sensors, and biosensors
Imaging and Analytical Interfaces	Integrated systems for real-time imaging and analysis of cells and tissues	Visualize cell behavior, track cell proliferation, and monitor drug responses	Fluorescence microscopy, confocal microscopy, and other imaging methods
Drug Delivery Systems	Mechanisms to introduce therapeutic agents or other substances to the cell culture chambers	Test the effects of drugs on tumor cells in a controlled manner	Microinjectors, diffusion-based systems, and integrated reservoirs
Interfacing Components	Connections to external equipment such as microscopes, pumps, and computers	Facilitate integration with analytical instruments and control systems for data acquisition and analysis	Microfluidic connectors, electrical interfaces, and data ports

Microfluidics in ToC devices allows for precise fluid control, enabling the recreation of TME conditions, such as perfusion rates for shear stress, nutrient gradients, differential diffusion rates of stimuli and cellular factors, and oxygen levels (Antoine et al., 2015; NDong et al., 2015).

#### 3.5.1 Applications in cancer modeling and drug screening

The ability of ToC to replicate the key features of TME (Mastrangeli et al., 2019; Mittal et al., 2019; Park et al., 2019; Thompson et al., 2020; Vulto and Joore, 2021; Dsouza et al., 2022; Li et al., 2023), holds great potential in disease modeling, drug screening, and personalized medicine. These devices enable researchers to conduct experiments under physiologically relevant conditions, accelerating the drug discovery process and enhancing the predictive power of preclinical studies (Table 5) (Hachey and Hughes, 2018).

**TABLE 5:** Main applications of ToC in the fields of cancer modeling, drug screening, and personalized medicine.

Cancer modeling	Drug screening	Personalized medicine
Metastasis/invasion studies	PK-PD modeling	PDOs
Tumor-heterogeneity	Toxicity	Biomarker discovery/validation
Cell-matrix interactions	High-throughput testing	Individual trials on-chip
Mechanical forces	Efficacy studies	Immunotherapy
Metastatic niche	Tumor resistance/sensitivity	Tailored clinical management
	Mechanistic testing	iPSCs
	Novel compound validation	Biopsy, blood or tissue-derived cells

Regarding ToC devices applied for cancer modeling, most studies are focused on assessing the impact of TME in metastasis (Portillo-Lara and Annabi, 2016; Hachey and Hughes, 2018), invasion processes (Wong et al., 2017), tumor heterogeneity (Acosta et al., 2014) and cell-matrix interactions (Zervantonakis et al., 2012; Zhao Y. et al., 2015). Such studies are performed by controlling different physiological parameters such as hypoxic gradients, ECM composition, and shear force. As models, co-cultures of cancer cells with inflammatory cells (e.g., macrophages), CAFs, and endothelial cells are typically performed in ToC applied to cancer modeling given its pivotal role in the onset of tumor progression (Sung et al., 2011; Zervantonakis et al., 2012; Sleeboom et al., 2018).

Besides cancer modeling, ToC devices can also be designed to offer a high-throughput platform for drug screening, allowing researchers to test multiple drugs and dosages, simultaneously. This is particularly useful for identifying potential drug candidates in a quick and automated manner. In fact, ToC has been described as a more reliable alternative for drug screening (Sun et al., 2017; Zhang et al., 2018; Dhiman et al., 2019), new drug validation (Gao et al., 2021), resistance (Rosa et al., 2014; Patel et al., 2015) and toxicity studies (Chen et al., 2015; Jeong et al., 2016). Several studies in the fields of drug screening, biomarker discovery, and immunotherapy testing have been conducted using ToC (Table 6). The results showed improved predictability, ability to high-throughput, and better insights regarding the mechanisms of drug activity and tumor responses (Mazzocchi et al., 2018; Chi et al., 2020; Skardal et al., 2020). Overall, this highlights the potential of ToC platforms for drug screening and improving the success rate of new compounds when advanced to clinical trials (Kim et al., 2012; Santo et al., 2017; Zhang et al., 2018; Dhiman et al., 2019).

**TABLE 6:** Examples of ToC applications in drug screening, cancer research and personalized medicine.

Application	Device type	Model	Target/drug in study	Ref
Drug Screening and Toxicity	Microfluidic array	MCF-7 Breast tumor spheroids	Doxorubicin and 4-hydroxytamoxifen	Prince et al. (2022)
	Collagen Matrix-Incorporated Microfluidic Chip	Co-culture spheroids of HT-29 Colon cancer and CAFs	Doxorubicin and paclitaxel	Jeong et al. (2016)
	Droplet-based microfluidics in PDMS device	Jurkat E6.1 cells, MDA-MB-231 cells and primary cells from tumor tissue	Bortezomib and Vorinostat for Jurkat cells Cisplatin and Epirubicin	Wong et al. (2017)
Modeling TME	Microfluidic system of PDMS with 3D gel scaffold	HT1080 Human fibrosarcoma cancer cells, MDA231 Breast carcinoma, Primary MVEC and HUVEC, macrophages and EC	Macrophage-mediated cancer cell invasion	Zervantonakis et al. (2012)
	Microfluidic co-culture chip for real-time integrative assays	Transitional cell carcinoma of the bladder (TCCB) and macrophages	Influence of lactate shuttling on the functional polarization and spatial distribution carcinoma cells and macrophages	Zhao Y. et al. (2015)
Modeling cell–matrix interactions	Microfluidic device with 3D hydrogel-based matrices	SUM-159 Breast carcinoma cells with Matrigel^®^, collagen I	Recapitulate 3D tumor-stroma interactions for studies of cell invasion and morphological changes	Truong et al. (2016)
	Microfluidic chip with electrospun Polycaprolactone (PCL) matrices	MDA-MB-231 breast cancer cells	Effect of different matrices in the 3D migration of MDA-MB-231 breast cancer cells	Eslami Amirabadi et al. (2017)
Modeling intrinsic physiological parameters	Microfluidic device in PDMS	PANC-1 Pancreatic adenocarcinoma cells	Effect of spatial oxygen gradients of chronic and dynamic hypoxic microenvironments in long term culture of cells	Acosta et al. (2014)
	Microfluidic device in PDMS	MRC-5 Human lung fibroblast and CL1-0 Human lung adenocarcinoma cells	Mimic the tensile strain that lungs are subjected to during breathing cycles to study tissue deformation	Huang et al. (2013)
Modeling tumor heterogeneity	3D bioprinted device with hydrogel matrix	MCF7 and MDA-MB-231 Breast cancer cells and MCF10A non-tumorigenic mammary epithelial cells	Creation of diverse tumor architectures that are representative of those found in various patients	Moghimi et al. (2023)
	Microfluidic device designed and fabricated at AIM BIOTECH	Human-derived spheroids from breast cancer individuals	Assess the effect of inter and intra-heterogeneity of spheroids in cell viability and migration behavior	Jeibouei et al. (2024)

#### 3.5.2 Applications in personalized therapy and precision medicine

ToC technology enables the customization of devices to reflect the unique characteristics of individual patient tumors, rendering it extremely advantageous for personalized medicine (Hachey and Hughes, 2018). The former aims to identify the most effective and least toxic therapy for each patient, unlike the “one-size-fits-all” approach used on standard oncology drugs, which often results in low efficacy and significant side effects (Ashley, 2016). This low efficacy hampers FDA approval of new drugs, and even approved drugs typically offer only marginal survival benefits (DiMasi et al., 2016).

Personalized medicine emphasizes the need for molecular testing on patient-derived samples to identify specific genomic aberrations and categorize cancer subtypes based on population-level genetics (Izumchenko et al., 2017). These tests allow informed treatment decisions, treating patients based on the most prominent characteristics of their tumor cohort (Chen et al., 2016; Izumchenko et al., 2017; Hachey and Hughes, 2018). However, accurately modeling the key characteristics of actual tumors has been challenging due to the limitations of established cancer cell lines (Ben-David et al., 2017).

Effective targeted therapies require models with high clinical predictive value, which is where ToC technology excels. By using patient-derived cells and tissues, ToC devices create personalized models for testing therapeutic responses and tailoring treatment strategies (Mathur et al., 2015; Huebsch et al., 2016; Takebe et al., 2017). Chip-based tumor models incorporating tissue samples from resistant sites, like primary and metastatic lesions are invaluable for studying tumor evolution and developing treatments that address tumor heterogeneity (Lancaster and Knoblich, 2014; van de Wetering et al., 2015; Bruna et al., 2016; Rios and Clevers, 2018).

Several cell sources can be exploited in chip-based personalized medicine, including primary cells from surgical resections, biopsies, aspirates, and blood samples (Papapetrou, 2016). From stem cell advances, organoid technology was developed, allowing it to retain genetic heterogeneity and mimic *in vivo* treatment responses of tumors. Still, organoids lack key anatomical features of tumors, like tissue-tissue interfaces, vascular compartments, dynamic fluid flow, and mechanical forces. Integrating organoids into chip-based models may better mimic organ-specific structures and gene expression (Lancaster and Knoblich, 2014; Rios and Clevers, 2018; Vlachogiannis et al., 2018).

Alternatively, cancer stem cells can be selected and differentiated on-chip, allowing to recreate native cell populations. This approach allows testing drugs on both healthy and tumor tissues from the same patient, which is ideal for accurate cancer modeling, drug testing and detecting treatment response variants (Burridge et al., 2016; Fong et al., 2016; Ronaldson-Bouchard and Vunjak-Novakovic, 2018). Nevertheless, generating healthy organoids for drug toxicity screening is impractical, requiring the use of patient-specific iPSCs (Hachey and Hughes, 2018).

## 4 Chips for the future

To overcome the current barriers in personalized medicine, ToC systems could be applied to cultivate patient’s cells (Haque et al., 2022; Zeng et al., 2023; Maulana et al., 2024). This approach allows high-throughput drug testing directly on an “avatar-on-a-chip,” predicting the patient’s response to various therapies. The results from these models can guide oncologists to the most effective treatments, a concept called two-way personalized medicine. This method aims to identify the best therapeutic regimen for a patient’s specific cancer before any treatment begins, offering a personalized drug screening tool for clinical use (Supplementary Table S2) (Kang et al., 2008; Caballero et al., 2017).

Individualized tumor chips can also facilitate “micro”-clinical trials, establishing multiple patient-specific tumor chips using cells and materials derived from each patient. These *in vitro* trials enable the testing of various drug doses and schedules, guiding treatment timing, and combination therapies, and discovering new agents (Supplementary Table S2). Drugs approved for specific cancers or those not traditionally used for cancer can be tested and potentially repurposed based on the results obtained on-chip, increasing treatment options and enhancing our understanding of cancer resistance mechanisms (Esch et al., 2015; Caballero et al., 2017).

ToC technology has the potential to transform oncology drug development and clinical management by enabling the sorting of patients based on individual characteristics without large-scale clinical trials, reducing human risk. It also permits therapeutic screening for patient groups unsuitable for standard trials, such as those with comorbidities, rare cancers, recurrent diseases, or pediatric patients. High-throughput experiments, combined with molecular testing, can elucidate gene-drug interactions, stratify tumor subtypes, and inform clinical trials (Supplementary Table S2), allowing for more precise patient categorization based on TME characteristics (Peterson and Houghton, 2004; Izumchenko et al., 2016; Izumchenko et al., 2017; Hachey and Hughes, 2018).

### 4.1 Challenges and considerations

ToC technology is extremely valuable for personalized medicine, requiring only a few patient cells and providing rapid and automated results within a clinically relevant timeframe. Even so, there are some challenges requiring thoughtful attention, such as the difficulty in generating primary cultures, the limited availability of viable patient-derived cells post-analysis, and the need for homogeneous replicates to represent tumor heterogeneity for comparative studies (Bhatia and Ingber, 2014; Dhiman et al., 2019). Additionally, regulatory and logistical hurdles associated with the use of patient-derived cells raise ethical considerations, including issues related to informed consent and privacy. Several other technical challenges exist, such as the complexity of device fabrication; the need for standardized protocols to ensure the scalability and reproducibility of the devices; the lack of laboratory frameworks for integrating multiple cell types and components; and the intellectual property aspects associated with the fabrication and commercialization of the devices (Huh et al., 2013; Edington et al., 2018).

### 4.2 Future directions and innovations

Before ToC technology can be widely adopted in precision medicine, it must overcome those challenges and deliver consistent results. Given its interdisciplinary nature, researchers, physicians, and regulatory bodies need to make a joint effort to address the remaining challenges that have been preventing ToC models from becoming the future of cancer research and personalized medicine.

To do so, studies using ToC devices must be performed to validate the clinical relevance of these chip-based models as translational tools (Bhatia and Ingber, 2014; Dhiman et al., 2019). For that, co-clinical trials should be conducted, comparing drug responses in ToC models with patient outcomes. Drug manufacturers and research centers can integrate *in vitro* studies using patient-derived biopsy tissue with *in vivo* trials, demonstrating the potential of ToC to improve drug development and patient treatment success. Implement accurate phenotypic and genotypic profiling to ensure ToC models closely resemble the original patient tumors. Additionally, establishing biobanks of human primary tissues and fostering cooperation among investigators can enhance the use of chip-based models (Supplementary Table S2).

Given the fast pace of engineering, emerging technologies such as advanced microfabrication techniques, nanotechnology, and organ-on-chip platforms are poised to enhance the capabilities of ToC devices. Also, the integration of artificial intelligence (AI) and machine learning (ML) with ToC technology offers significant potential for data analysis and interpretation. AI and ML can help identify patterns, predict outcomes, and optimize experimental designs, thereby accelerating research and improving therapeutic strategies.

## 5 Conclusion

Developing effective anticancer therapies remains challenging due to the limitations of traditional models in accurately mimicking human tumor biology and treatment responses. Conventional 2D cell cultures and animal models fall short in replicating the complex TME, essential for understanding tumor behavior and therapeutic outcomes. This has led to the rise of advanced *in vitro* models like tumor spheroids and organoids, which better represent the 3D structure and biological complexity of *in vivo* tumors. These 3D models, by closely mimicking the TME, have been providing key insights into tumor biology and improving drug response predictability, thus facilitating the development of more reliable anticancer therapies. The integration of these models into microfluidic platforms further enhances their utility by allowing better control over physiological conditions and enabling high-throughput screening.

Effective targeted therapies require models with high clinical predictive value, which is where ToC technology excels. ToC devices use patient-derived cells and tissues to create personalized models for testing therapeutic responses and tailoring treatment strategies. These models, incorporating tissue samples from primary and metastatic lesions, are invaluable for studying tumor evolution and developing treatments that address tumor heterogeneity. ToC systems can be applied to cultivate patient cells, allowing high-throughput drug testing on an “avatar-on-a-chip” model, predicting patient responses to therapies. This two-way personalized medicine approach aims to identify the best therapeutic regimen for a patient’s specific cancer before treatment begins. Individualized tumor chips can also facilitate “micro”-clinical trials, testing various drug doses and schedules, and guiding treatment timing and combination therapies. However, the successful implementation of these models faces several challenges, including the need for standardized protocols, overcoming technical limitations, and addressing ethical and regulatory concerns.

Continuous advancements in microfabrication, tissue engineering, and the integration of artificial intelligence and machine learning are expected to enhance the capabilities of these models. These innovations will improve the accuracy, functionality, and scalability of 3D cancer models, accelerating their adoption in preclinical research and personalized medicine.

In conclusion, the development and exploitation of tumor spheroids, organoids, and ToC technology represent significant advancements in cancer modeling. These systems grant a more precise depiction of human-derived tumors, providing a powerful platform for drug development and personalized therapeutic strategies. As technology evolves, these models are poised to play a crucial role in the future of cancer research, leading to more effective treatments and improved patient outcomes.
