# Supplementary material for: Breaking the mold: 3D cell cultures reshaping the future of cancer research
Source: Front Cell Dev Biol. 2024 Nov 26;12:1507388. doi: 10.3389/fcell.2024.1507388 (PMC11628512; doi:10.3389/fcell.2024.1507388)
Supplement: Supplementary file 1 [file Table1.docx]

Supplementary Material

Breaking the Mold: 3D Cell Cultures Reshaping the Future of Cancer Research

Sandra Cordeiro^1,2†^, Beatriz B. Oliveira^1,2†^, Ruben Valente^1,2^, Daniela Ferreira^1,2^, André Luz^1,2^, Pedro V. Baptista^1,2*^, Alexandra R. Fernandes^1,2*^

^1^ UCIBIO, Dept. Ciências da Vida, Faculdade de Ciências e Tecnologia, Universidade NOVA de Lisboa, 2819-516 - Caparica, Portugal

^2^ i4HB, Associate Laboratory – Institute for Health and Bioeconomy, Faculdade de Ciências e Tecnologia, Universidade NOVA de Lisboa, 2819-516 - Caparica, Portugal

**^†^ Equal contribution**

*** Correspondence:**Pedro V. Baptista (PVB) [pmvb@fct.unl.pt](mailto:pmvb@fct.unl.pt)
Alexandra R. Fernandes (ARF) [ma.fernandes@fct.unl.pt](mailto:ma.fernandes@fct.unl.pt)

**Supplementary Table 1.** Examples of 3D models main applications in cancer research.

| **Application** | **Cancer type** | **Description** | **Advantages** | **Ref** |
| --- | --- | --- | --- | --- |
| **Drug development and screening** | Colorectal cancer (CRC) | The potential mechanisms of action of tetrac in CRC cells using a perfusion bellows cell culture system. | This innovative system facilitated drug screening, revealing insights into how drug resistance dynamics are influenced by cell status. | (Chin et al., 2019) |
|  |  | Explored an improved preclinical model and compared the genomic characteristics and DNA repair proficiency of CRC models to patients who responded to pharmaceuticals. | This methodology facilitated the discovery and confirmation of efficacious targeted drug combinations to investigate CRC. | (Arena et al., 2020) (Wang et al., 2020) |
|  |  | Tumor samples were collected from CRC patients to establish PDX models for drug screening to identify and validate effective targeted drug combinations. | Identification of synthetic lethal compounds associated with PTEN deficiency, through synthetic lethality drug screening of PTEN-isogenic CRC cells. | (Yao et al., 2020) |
|  |  | Characterization of an LS180 spheroid model for screening of anti-CRC compounds within alginate encapsulation solution. | These spheroids proved to be useful for assessing new chemotherapeutic compounds targeting CRC. | (Smit et al., 2020) |
| **Drug development and screening** | CRC | Recent advancements in CRC *in vitro* model systems, encompassing conventional organoids such as conditional reprogramming-based cell cultures, along with cutting-edge experimental models such as ToC platforms or liquid biopsy techniques. | This study suggested that hydro organoids possess potential for integration into high-throughput assays and drug screening models, potentially aiding in predicting clinical outcomes. | (Ramzy et al., 2020) |
|  |  | Drug sensitivity tests for subsequently administered the same chemotherapy to patients with rectal cancer. Drugs found to be effective in the PDOs were successful in 88% of patients. | These findings suggest the clinical relevance of PDOs in accurately predicting drug responses, thereby presenting the potential to guide individualized treatment strategies. | (Vlachogiannis et al., 2018) |
|  | Lung cancer (LC) | Lung carcinoma cell lines were used to create a 3D biosensor based on LC spheroids, employing interdigitated electrodes for evaluating drug efficacy. | The development of LC organoids has significantly advanced drug screening efforts, offering more accurate and personalized models for evaluating therapeutic efficacy. | (Wu et al., 2018) |
|  |  | Description of a new technique for establishing durable human airway organoids from broncho-alveolar resections or lavage material capable of long-term expansion. | These organoids maintain tumor histopathology and cancer related gene mutations, suggesting that they can be utilized effectively in drug screening assays. | (Sachs et al., 2019) |
|  |  | Development of LC organoids utilizing a distinct cell source-pleural effusion aspirate-obtained from multiple LC patient. | 3D hydrogel-based models possibly provides enhanced disease replication and characterization compared to standard 2D cell cultures, particularly when using patient-derived cells. | (Mazzocchi et al., 2019) |
|  |  | LC and normal bronchial PDOs, among others, were employed in an airway organoid culture system to successfully cultivate LC organoids. | The potential usefulness of this new system in high-throughput drug screening and *in vitro* models to predict drug response in patients, potentially can facilitating personalized cancer treatment strategies. | (Kim et al., 2019) |
|  | Ovarian cancer (OC) | Protocol enabling the effective derivation and prolonged expansion of organoids. | OC organoids more closely resemble the *in vivo* tumor microenvironment (TME) than conventional 2D cell culture for drug screening. | (Kopper et al., 2019) |
|  |  | Creation of patient-derived organoids (PDOs), focus on high-grade serous OC. | Pre-clinical tools for conducting personalized drug screening, highlighting the significance of the NRG1/ERBB pathway. | (Maenhoudt et al., 2020) |
|  |  | PDOs to predict clinical drug response and functional consequences of tumor heterogeneity. | PDOs drug screening revealed drug sensitivity in a substantial majority of OC patients. | (de Witte et al., 2020) |
| **Drug development and screening** | OC | Developing an efficient method for culturing organoids from both ovarian and endometrial tumor. | Improved models that accurately represent gynecologic malignancies. | (Maru et al., 2019) |
|  | Pancreatic ductal adenocarcinoma (PDAC) | Biomaterial substrate-derived dense cellular spheroids resembling the behavior of PDAC and it microenvironment. | Efficiently established a 3D tumor spheroid model for PDAC. | (Wong et al., 2019) |
|  |  | Aiming to expand the establishment of organoids derived from diverse cases of biliary tract and pancreatic cancers. | Facilitating personalized therapeutic approaches for individual patient. | (Saito, 2019) |
|  |  | Their approach revealed protein arginine methyltransferase gene 5 (PRMT5) as a promising druggable target. | Unbiased genetic screening in combination with a clinically relevant model system for identifying effective synthetic lethal drug combinations. | (Camara et al., 2020) (Wei et al., 2020) |
|  |  | A study evaluating 76 drugs across 30 PDOs obtained from PDAC revealed the potential of the PRMT5 inhibitor EZP015556 as a drug capable of inhibiting MTAP-negative tumors. | These organoids are cryopreservable and serve as a dynamic biobank of PDAC cells for both identifying biomarkers and serves as a valuable platform for drug screening. | (Driehuis et al., 2020) |
|  |  | Research involved establishing 66 patient-derived xenografts (PDXs) emphasizing the importance of understanding the potential mechanisms driving therapeutic resistance. | Identification of potential biomarkers and therapeutic targets can possibly lead to the discovery of novel therapeutic targets and the development of combination therapies. | (Yang et al., 2021) |
|  | Prostate cancer (PC) | An innovative system enables a high-throughput screening of 2,427 drugs, utilizing PDOs. The study specifically focuses on CRC organoids, which exhibit diverse sensitivities to these compounds. | These findings lay a solid foundation for the development of personalized therapeutic approaches in PC. | (Kondo and Inoue, 2019) |
|  |  | Diagnostic leukapheresis was utilized to isolate CTCs from metastatic PC patients, then these CTCs were cultured as organoids. | These PDOs serve as models for drug discovery and sensitivity screening, aiming to explore potential treatment options. | (Mout et al., 2021) (Metastatic Prostate Cancer, 2018) |
| **Personalized medicine** | Bladder cancer | High-throughput screening, focused on the interaction between PDOs and tumor-specific cytotoxic T cells. | Identification of three epigenetic inhibitors: BML- 210, GSK-LSD1, and CUDC-10; demonstrating significant antitumor effects, which BML-210 exhibited a remarkable ability to enhance sensitivity to a PD-1 inhibitor. | (Zhou et al., 2021) |
| **Personalized medicine** | Bladder cancer | PDOs cocultured CAR-T cells targeting MUC1 spontaneously migrated toward MUC1+ cells and induced a specific cell lysis. | Bladder cancer PDOs recapitulate the heterogeneity and key features of parental cancer tissues and could be useful for preclinical testing of CAR-T cells *in vitro*. | (Yu et al., 2021) |
|  | Bladder cancer and Glioblastoma | Development of organoid models in coculturing experiments by introducing MUC1-CAR-T cells and EGFR VIII-CAR-T cells to their respective tumor organoids. | Prove the degree of preservation of tumor organoids on biological characteristics of primary tumor tissue and verify the anti-tumorigenic effect of these innovative CAR-T cell targets. | (Yu et al., 2021a) (Jacob et al., 2020a) |
|  | Breast cancer (BC) | A microfluidic BC cell spheroid model was developed to study natural killer (NK) cell immunotherapies. NK cells were able to directly penetrate the spheroids and destroy tumor cells. | NK cells could be genetically engineered to express CAR to increase their cytotoxicity function and to guide them toward tumors. | (Ayuso et al., 2019) (Xie et al., 2020) |
|  | CRC | Researchers tested EGFR and MEK inhibitors on a large panel of CRR organoids in order to determine the effect of the Ras-mutation status on the sensitivity to these drugs. | CRISPR/Cas9 technique could remove oncogenic mutations, thus enabling CRR treatment. They demonstrated that the introduction of a KRASG12D mutation resulted in loss of drug sensitivity compared to WT. | (El Harane et al., 2023) |
|  |  | A platform was also designed for the assessment of CAR-NK-92-mediated activity against PDOs from CRC. | A sensitive *in vitro* platform to evaluate CAR efficacy and tumor specificity in a personalized manner. For example, CAR- engineered NK-92 cells were directed toward tumor organoids. | (Schnalzger et al., 2019) |
|  | Gastric cancer (GC) | Organoids of human and mouse GC to simulate the typical features of human gastric cancer and the pathway of abnormal activation signal pathway change. Through drug screening, they identified organoids with different responses to conventional chemotherapy drugs | The successful interference of drugs (e.g., 5-fluorouracil (5-FU), irinotecan, epirubicin, oxaliplatin and docetaxel)  with abnormal activation pathways in demonstrates the potential use of organoids as models for testing therapeutic response. | (Kita et al., 2019) |
|  |  | Establishment of GC organoids and successfully acquired 5-FU-resistant GC organoids. | This finding highlights KHDRBS3 as a potential biomarker for predicting treatment outcomes and prognosis in patients with GC. | (Ukai et al., 2020) |
|  | Glioblastoma | Glioblastoma organoids to recapitulate inter- and intra-tumoral heterogeneity could be employed to test CAR-T cell immunotherapy in a clinically relevant timescale. | EGFRvIII-specific CAR-T cells were shown to penetrate organoids and a higher expansion of these cells was observed was accompanied by increased cytotoxicity | (Jacob et al., 2020) |
| **Personalized medicine** | Glioblastoma | Engineered NK-CAR cells targeting HER2, EGFR, and EGFRvIII were investigated in 3D tumors. | These models presented a dispersed morphology with an increase in the number of dead cells and in INF-γ, IL-6, and IL-8 secretion when cocultured with the super-charged NK cells. | (Xie et al., 2020) |
|  | Glioma | Glioma organoids and human umbilical vein endothelial cells in a coculture system within a fibrin gel, the drug atorvastatin exhibited notable inhibitory effects on angiogenesis, which were found to be dose-dependent. | Downregulation of VEGF, CD31, and Bcl-2. This phenomenon suggests that atorvastatin holds promising potential as an agent for the treatment of cancer. | (Bayat et al., 2018) |
|  | Head and neck cancer | PDOs from head and neck cancer were exposed to chemotherapy, radiotherapy and targeted drugs. Also, organoid gene editing based on CRISPR-Cas9 has been applied to biomarker validation. | The results showed that there was a significant correlation between organoid sensitivity and clinical response. They show how organoids can be used to explore biomarker potential in targeted therapeutic settings. | (Millen et al., 2023) |
|  | Leukemia and Head and neck squamous cell carcinoma | Individuals with congenital dyskeratosis, caused by mutations in the DKC1 gene, have a predisposition to developing leukemia and squamous cell carcinoma of the head and neck. | hPSC-derived intestinal organoids from patients with this disease have been developed and used for Cas9-mediated recombination, and mutation correction resulted in a phenotypic rescue | (Woo et al., 2016) |
|  | Multicellular hepatocellular carcinoma (HCC) | These organoids incorporate multicellular HCC cells along with various stromal cells (e.g., endothelial cells, fibroblasts, and hepatic stellate cells). | Strong association between the activation of Yes-associated protein/transcriptional coactivator with PDZ-binding motif signaling and stromal activation in multicellular HCC. | (Cho et al., 2021) |
|  | Non-small cell lung cancer (NSCLC) | Cocultured tumor organoids expressing B7-H3 with B7-H3-CAR-T cells to verify their antitumor activity prior to brain metastasis. | PDX model that is able to bind with CCR2 on the surface of tumor cells can promote CCR2b-B7-H3-CAR-T cells to penetrate the blood–brain barrier. | (Li et al., 2022) |
|  |  | Preliminary investigation to examine the safety and antitumor efficacy of pyrotinib using PDOs and corresponding PDXs from patients with advanced lung adenocarcinoma characterized by the presence of the HER2-A775_G776YVMA insertion. | Pyrotinib exhibits substantial growth inhibition of organoids and induces a notable reduction in tumor burden within PDX models. Also, a phase II clinical trial involving 15 patients with HER2-mutant NSCLC demonstrated the therapeutic advantages of pyrotinib. | (Wang et al., 2019) |
|  | OC | High-grade serous tubo-OC organoids were obtained with mouse fallopian epithelial cells using a CRISPR/Cas9 mutagenesis. | These organoids presented several expected sensitivities to small molecule drugs, particularly PARP inhibitors | (Zhang et al., 2021) |
| **Personalized medicine** | OC | OC spheroids were also subjected to genome editing using CRISPR/Cas9 to inactivate TIMP-2, with the goal to understand its pathogenic role. | These KO spheroids exhibited enhanced proliferation, migration, invasion, and resistance to paclitaxel | (Escalona et al., 2022) |
|  |  | Customized therapies targeting ALDH1 could reduce resistance to chemotherapy and improve the survival rate of OC. | ALDH1 inhibition by CRISPR/Cas9 effectively blocked the proliferation and survival of OC spheroids | (Kim et al., 2018) |
|  |  | A microdevice platform that recapitulated a 3D ovarian tumor allowed the exposition of tumor cells to CAR-T cell delivery and revealed cytotoxicity. | This design allows for the evaluation of CAR-T cell cytotoxicity and infiltration in the heterogeneous oxygen landscape of *in vivo* solid tumors at a previously unachievable scale *in vitro*. | (Ando et al., 2019) |
|  | PDAC | A group generated PDAC organoids and found that Pin1 is overexpressed both in cancer cells and CAFs and correlates with poor survival. | Targeting Pin1 function by CRISPR/Cas9 suppressed fibroblast proliferation, induced quiescence, and inhibited their ability to secrete a wide range of cytokines that promote cancer progression and prevent T cell recruitment into the TME. | (Koikawa et al., 2021) |
|  | PDAC, CRC and GC | Gene editing of KRAS and TP53 through CRISPR/Cas9 was possible in PDOs from PDAC, CRR, and GC, and this correction inhibited PDOs growth. | Programmable nucleases, particularly the CRISPR/Cas9 system, provide an attractive tool for genetically targeting KRAS mutations in cancer cells. | (Sayed et al., 2022) |

**Supplementary Table 2.** Complete and on-going clinical trials using organoids and TOCs systems (according to clinicaltrials.gov, accessed on October 3^rd^, 2024).

| **Model/System** | **Cancer type** | **Study title** | **Description** | **NCT number** |
| --- | --- | --- | --- | --- |
| **Organoids** | Biliary tract cancer | Trifluridine/Tipiracil and Irinotecan for the Treatment of Advanced Refractory Biliary Tract Cancer | PDOs for drug screening and correlation with clinical response to trifluridine/tipiracil plus irinotecan. | NCT04072445 |
|  |  | Feasibility Study of Multi-Platform Profiling of Resected Biliary Tract Cancer | Development of a multi-Platform Profiling using PDOs for drug sensitivity screening. | NCT04561453 |
|  | Bladder cancer | Urothelial Cancer Tumor Biomarkers and Physical-spectroscopic Characteristic | PDOs to characterize biomolecular, proteomic, metabolomic and spectroscopy of urothelial bladder neoplasia. | NCT04770974 |
|  |  | Precise Neoadjuvant Chemoresection of Low Grade NMIBC | Generation of PDOs for drug screening to select the appropriate anticancer agents in PDOs. | NCT06227065 |
|  |  | Guiding Instillation in Non Muscle-invasive Bladder Cancer Based on Drug Screens in PDOs | Drug sensitivity tests in PDOs to establish drug screening in clinical settings and clinical trials. | NCT05024734 |
|  | Bone marrow cancer | Novel 3D Hematological Malignancy Organoid Platform to Study Disease Biology and Perform Chemosensitivity Assays for Patient-Specific Care | Drug screening and investigation of the mechanism of resistance in myeloma PDOs. | NCT03890614 |
|  | BC | Developing Breast (Cancer) Organoids | Creation of breast PDOs biobank. | NCT05317221 |
|  |  | Clinical Study on Drug Sensitivity Verification or Prediction of Therapy for BC by PDO Model | Drug screening and personalized medicine in BC PDOs. | NCT03544047 |
|  |  | BC Subtype Characterization Through PDOs | Personalized medicine using breast PDOs. | NCT06315868 |
|  |  | Biomarkers and Clinical Features of Metastatic BC in Patients Treated with CDK4/6 Inhibitors | Development of PDOs to evaluate mechanisms of resistance and response to treatment. | NCT04526587 |
|  |  | Consistency of Organoids Based Drug Sensitivity and Efficacy of Neoadjuvant Chemotherapy in BC | Evaluation of the sensitivity of chemotherapy regime in BC PDOs | NCT06155305 |
|  |  | Organoid Model Predictive of Response to Immunotherapies | Research of novel therapies for BC using PDOs models. | NCT06084676 |
|  |  | Markers to Evaluate the Efficacy of PH-based Regimen as a Neoadjuvant Therapy for Operable HER2 Positive BC | Establishing PDOs for drug sensitivity assays. | NCT04281641 |
|  |  | Academia Sinica Investigator Award 2010 | Evaluation of anticancer phytocompounds in PDOs to study cancer progression and metastasis. | NCT01287468 |
| **Organoids** | BC | A Pilot Study of a Micro-Organosphere Drug Screen Platform to Lead Care in Advanced BC | Generation of patient-derived micro-organospheres to determine sensitivity to the most common chemotherapy agents used in advanced BC treatment. | NCT04655573 |
|  |  | UZ/KU Leuven Program for Post-mortem Tissue Donation to Enhance Research | PDOs and PDXs to unravel metastatic BC evolution, biology, hereditary cancer syndromes, heterogeneity and treatment resistance. | NCT04531696 |
|  |  | Collection of Specimens and Clinical Data for Patients with Recurrent or Metastatic BC or Male BC | PDOs and PDXs to evaluate response to targeted therapies. | NCT06217874 |
|  |  | Precise Therapy for Refractory HER2 Positive Advanced BC | PDOs divide into six groups according to genomic signatures for personalized medicine. | NCT05429684 |
|  |  | De-convoluting Interactions Between Genes, the Cancer Environment, and the Immune System to Develop Therapies That Work for You | PDOs to perform functional genomics experiments and to determine if the cancer cells can be reprogrammed using radiotherapy and other interventions. | NCT05134779 |
|  |  | Functional Precision Oncology to Predict, Prevent, and Treat Early Metastatic Recurrence of TNBC | PDXs and PDOs for drug profiling while patient is undergoing preoperative chemotherapy, surgery and radiotherapy. | NCT05464082 |
|  |  | Prospective Study to Correlate the Treatment Sensitivity of PDOs with Clinical Outcomes in BC Patients with Brain and/or Extra-cranial Metastases | Personalized medicine (assessing radio-sensitivity and chemo-sensitivity) utilizing resected brain or resected/biopsied extra-cranial metastases of BC PDOs | NCT06468124 |
|  |  | Sensitivity Detection and Drug Resistance Mechanism of BC Therapeutic Drugs Based on Organ-like Culture | Drug screening and evaluation of possible mechanisms of BC drug resistance in BC PDOs. | NCT03925233 |
|  |  | [18F]Fluoroestradiol-PET/CT Companion Imaging Study to the FORESEE Trial | PDOs with ER status for assessment of FES-PET/CT imaging and for drug profiling results to predict response to hormonal therapies. | NCT04727632 |
|  |  | Personalized Patient Derived Xenograft (pPDX) Modeling to Test Drug Response in Matching Host | Molecular Profiling and drug testing in organoid cultures and *in vivo* pPDX. | NCT02732860 |
|  |  | Clinical Treatment of Refractory BC Based on Organoid Drug Sensitivity Results | Drug screening in validated BC PDOs and development of personalized treatment plans. | NCT06438055 |
|  |  | Functional Precision Oncology for Metastatic BC | Development of PDOs for drug screening and personalized medicine. | NCT04450706 |
|  |  | Development of PDXs in Patients with BC | PDXs and PDOs to improve BC personalized treatment. | NCT04703244 |
|  |  | Real Time Molecular Analysis of BC Receiving Neo-adjuvant Chemotherapy | PDOs for mimicking patient's response neo-adjuvant chemotherapy | NCT04504747 |
| **Organoids** | BC | Biomarkers of Efficacy and Tolerability of Sacituzumab-Govitecan in the Treatment of Patients with Triple-negative BC in the Metastatic Phase: Prospective Multicenter Real-world Study | Development organoids derived from different subgroups of patients to analyze biomarkers and response to treatment. | NCT06240195 |
|  |  | Development of Multi-specific Antibodies Based on Immune Microenvironment of BC | PDXs and PDOs to study the TME of BC patients and to design multi-specific antibody drugs to improve the efficacy of immunotherapy. | NCT05767931 |
|  |  | PDO-guided Personalized Treatment Versus Treatment of Physician's Choice in Patients with Relapsed and Refractory BC: a Multicenter, Randomized, Controlled Phase III Trial | Generation of BC PDOs for drug screening aiming the comparation of guided personalized treatment and treatment chosen by physicians. | NCT06268652 |
|  |  | Cohort Study to Determine the Antitumor Activity of New CAR-macrophages in BC PDOs (CARMA) | Evaluation of the antitumor activity of new CAR-macrophages therapy in BC PDOs. | NCT05007379 |
|  |  | Establishment of an *ex vivo* Tumor Collection of Triple-negative BC in Order to Validate the Interest of Innovative Therapies and the Search for Predictive Biomarkers of Response to Treatment | Generation of validated triple-negative BC PDOs for the establishment of a biobank, aiming to spark the interests of these models for development of novel therapies and identification of specific biomarkers. | NCT05404321 |
|  |  | Organoid-Guided Functional Precision Therapy Versus Treatment of Physician's Choice in Previously Treated HER2-negative Advanced BC: a Phase II, Multicenter, Open-label, Randomized Controlled Trial | Generation of HER2-negative locally advanced or metastatic BC PDOs for drug screening and personalized treatment strategies aiming the translation to the clinic. | NCT06102824 |
|  |  | Quadratic Phenotypic Optimization Platform (QPOP) Utilization to Enhance Selection of Patient Therapy Through PDOs in BC | Buildout of a QPOP platform able to identify patient-specific drug combinations across a range of BC PDOs for guided therapy in patients. | NCT05177432 |
|  | BC with liver metastasis | Clinical, Histological and In-depth Molecular Characterization as Well as Experimental Models of Liver Metastasis from Patients with BC in Order to Identify New Potential Treatment Avenues | PDOs and PDXs derived from liver metastasis to characterize the tumor cells and TME at the transcriptomic and protein single-cell level. | NCT05720676 |
|  | BC with metastasis | Efficacy of Personalized Tumorogram-based Therapy in Cancer Established from Patient-derived Biological Avatar: Proof-of-concept Study | Institution of an informative tumorogram for treatment recommendation, including drug screening and personalized therapies data obtained by using metastatic BC PDOs. | NCT06459791 |
| **Organoids** | Cholangiocarcinoma | Study on Consistency Evaluation for Drug Sensitivity of PDO Model from Cholangiocarcinoma Patients | PDOs model to predict the clinical chemotherapeutic efficacy and the possibility of guiding the adjuvant chemotherapy. | NCT05634694 |
|  | CRC | Intestinal Organoids | Generation of CRC organoids for the screening of potential therapeutic molecules. | NCT05294107 |
|  |  | Feasibility of Establishing PDOs for Rectal Cancer: A Biospecimen Collection Protocol | Establishment of rectal PDOs as disease models. | NCT04371198 |
|  |  | Validation of Organoids Potential Use as a Companion Diagnostic in Predicting Neoadjuvant Chemoradiation Sensitivity in Locally Advanced Rectal Cancer | Rectal PDOs generation and evaluation of organoids response to irradiation and chemotherapy. | NCT03577808 |
|  |  | Testing ONC201 to Prevent CRC | Establishment of PDOs *ex vivo* and compare adenoma-derived organoid take rates between samples obtained prior to and following treatment. | NCT05630794 |
|  |  | Early-Line Anti-EGFR Therapy to Facilitate Retreatment for Select Patients With mCRC | PDOs to predict clinical response to EGFR inhibition. | NCT04587128 |
|  |  | Precision Chemotherapy Based on Organoid for CRC PDOs Drug Sensitivity for CRC: A Prospective, Multicentre, Randomized, Controlled Trial | Drug screening utilizing state IV CRC PDOs models. | NCT05832398 |
|  |  | Precision Medicine for Advanced or Recurrent CRC Directed by High-throughput Sequencing and Tumor Organoids Model | Molecular profiling and establish of CRC PDOs and PDXs aiming drug screening, discovery of new molecular targets and personalized medicine. | NCT05883683 |
|  |  | PDOs of RAS/RAF Wild-type Metastatic Right Colon Cancer to Test the Sensitivity and Clinical Consistency of Combined Treatment of Cetuximab | Drug screening employing RAS/RAF wild-type metastatic right colon cancer PDOs. | NCT04906733 |
|  |  | Future of CRC Surgery | PDOs to examinate *in vitro* tumor uptake and distribution of fluorescence agents. | NCT04220242 |
|  |  | Dissecting the Biology of Early-onset CRC | 3D spheroids and organoids to investigate clonogenic activity, tumorigenic/invasive capacity, and to analyze mechanisms involving TME components. | NCT05916443 |
|  |  | Manipulation of the Gut Microbiome by a Standardized Preoperative Diet to Prevent CRC Recurrence and Metastasis Following Surgery | Culture of PDOs to study the impact of diet in patients to prevent CRC recurrence or metastasis. | NCT06349590 |
| **Organoids** | CRC | Imatinib as Pre-operative Anti-Colon Cancer Targeted Therapy | Effects of imatinib on the ability of cancer cells to form *in vitro* PDOs. | NCT02685046 |
|  |  | Systemic Neoadjuvant and Adjuvant Control by Precision Medicine in Rectal Cancer | PDOs treatment response to compare to their corresponding clinical and pathological response. | NCT04842006 |
|  |  | A Real-world Observational Clinical Study Aims to Assess the Consistency of Clinical Efficacy in CRC Treatment and Drug Susceptibility Outcomes Using a Novel Drug Susceptibility Testing Method | Novel drug screening method investigation using CRC PDOs models. | NCT06100016 |
|  |  | The Theranostic Value of STARD3 in CRC: The STAR Study | Establish PDOs to explore STARD3 role as a dynamic biomarker of treatment response and drug sensitivity. | NCT06136949 |
|  |  | Drug Screening of PDOs from Advanced/Recurrent/Metastatic CRC Culture to Personalized Therapy, an Exploratory Research | Drug screening for validation in advanced/recurrent/metastatic PDOs, through next generation sequencing (NGS) and histopathology analysis. | NCT05304741 |
|  |  | The Clinical Efficacy of Drug Sensitive Neoadjuvant Chemotherapy Based on Organoid Versus Traditional Neoadjuvant Chemotherapy in Advanced Rectal Cancer | Development of personalized neoadjuvant therapy combined with drug screening methods using rectal cancer PDOs. | NCT05352165 |
|  |  | Tumor Immune Microenvironment Involvement in CRC Chemoresistance Mechanisms | Evaluation of anticancer therapies using PDOs. | NCT05038358 |
|  |  | Exosomes in Rectal Cancer | Mouse models and PDOs to measure the functionality of exosomes. | NCT03874559 |
|  |  | Study and Modulation of Immune Responses in Primary and Metastatic Colon Cancers | Coculture of PDOs to study immune responses of TME for immunotherapy. | NCT06435689 |
|  |  | Study to Investigate Outcome of Individualized Treatment in Patients with Metastatic CRC | PDOs for *ex vivo* drug sensitivity testing and personalized medicine. | NCT05725200 |
|  |  | Tailoring Treatment in CRC | Drug screening of PDO to identify effective target therapies for personalized CRC treatment. | NCT05401318 |
|  |  | Test of CD47-SIRPα Inhibitors on the Immune Microenvironment Colon Cancer | Assessment of complex tumor immune interaction for the evaluation of immunotherapies using PDOs. | NCT05955196 |
| **Organoids** | CRC | Validation of the Three-dimensional Bioprinted Tumor Models as a Predictive Method of the Response to Chemotherapy for CRC with or without Liver Metastases | Establishment and validation of 3D bioprinted CRC PDOs for drug screening evaluation. | NCT04755907 |
|  | CRC and HCC | Prospective Clinical Validation of NGS and PDOs Guided Therapy in Patients with Advanced/ Inoperable Solid Tumors | Drug screening and personalized medicine using NGS validated PDOs. | NCT06077591 |
|  | CRC and liver metastasis | The Exploratory Study of PDOs for the Prediction and Evaluation of Clinical Efficiency Effect of CRC Liver Metastasis | Development of CRC and liver metastasis PDOs focusing on drug screening applications. | NCT05183425 |
|  | Endometrial cancer | Collection of Endometrial Cancer-derived Organoids to Evaluate the Efficacy of PARP Inhibitors: PENDOR Pilot Study | Evaluation of the efficacy of cancer treatments based in PARP inhibitors in endometrial PDOs. | NCT06603506 |
|  | Esophageal cancer | Chemoradioresistance in Prospectively Isolated Cancer Stem Cells in Esophageal Cancer-Organoid: RARE STEM-Organoid | Esophageal cancer PDOs development aiming drug screening. | NCT03283527 |
|  |  | Molecular Characteristics of Gastroesophageal Adenocarcinoma (MOCHA): A Prospective Feasibility Study | Establishment of PDOs and PDXs models to identify drug sensitivity to guide treatment decisions. | NCT04219137 |
|  |  | Prediction Model of Response for CCRT in Esophageal Cancer | PDOs tretment with radiotherpay 5-FU and to evaluate chemoradiotherapy response. | NCT03081988 |
|  | Esophagogastric cancer | Tissue Collection Protocol for Gastroesophageal Cancers | PDOs culture to create a bank of primary esophageal/gastric cancers. | NCT02495337 |
|  |  | Liposomal iRInotecan, Carboplatin or oxaliplatin for Esophagogastric Cancer | Measurement of PDOs growth after treatment. | NCT03764553 |
|  |  | OPPOSITE: Outcome Prediction of Systemic Treatment in Esophagogastric Carcinoma | PDOs *in vitro* response to treatment correlation with the *in vivo* response of patients. Characterization of whole genome, methylome and RNA sequencing prior and after systemic treatment. | NCT03429816 |
|  | GC | A Prospective Observational Study on the Potential Benefit of Neoadjuvant Therapy for Advanced GC Based on Organoid Drug Susceptibility Screening | Drug screening of neoadjuvant chemotherapies in early GC PDOs. | NCT05442138 |
| **Organoids** | GC | Exploratory Study of GC Organoids in the Screening of Neoadjuvant Chemotherapy and Immunotherapy Drugs | Drug screening and personalized medicine evaluation in gastric PDOs. | NCT06196554 |
|  |  | A Real-world Observational Clinical Study Aims to Assess the Consistency of Clinical Efficacy in GC Treatment and Drug Susceptibility Outcomes Using a Novel Drug Susceptibility Testing Method | Novel drug screening method investigation using GC PDOs. | NCT06100003 |
|  |  | Consistency Between Treatment Responses in PDOs Models and Clinical Outcomes of Neoadjuvant Therapy, Conversion Therapy and Palliative Therapy in GC | Investigation of neoadjuvant therapy, conversion therapy and palliative chemotherapy effectiveness employing gastric PDOs. | NCT05203549 |
|  |  | The Clinical Efficacy of Drug Sensitive Neoadjuvant Chemotherapy Based on Organoid Versus Traditional Neoadjuvant Chemotherapy in Advanced GC | Assessment of safety and clinical value of the personalized neoadjuvant therapy based on PDOs organoid drug sensitivity assay. | NCT05351398 |
|  | GC and gastroesophageal junction adenocarcinoma | Biomarker Analysis of Tislelizumab Combined with Chemotherapy for Perioperative Treatment of G/GEJ Adenocarcinoma | PDOs treatment with immune-chemotherapy drugs to predict drug responses to corresponding patients who accepted neoadjuvant therapy. | NCT05508399 |
|  | Gastro-entero-pancreatic neoplasms | Generation of Organoids of Neuroendocrine Neoplasms of the Gastro-Entero-Pancreatic Tract Obtained from Patients Undergoing Surgery | Establishment of gastro-entero-pancreatic neoplasms PDOs as viable disease models. | NCT06519500 |
|  | Gastrointestinal cancer | The Innervation of Human Gut Sensory Epithelial Cells | Gastrointestinal PDOs used to study the biology of innervated sensory epithelial cells. | NCT02888587 |
|  |  | Research on the Correlation Between Organoid Drug Sensitivity Testing and Precise Treatment of Gastrointestinal Tumors | Gastrointestinal PDOs development for drug screening and investigation of new methods for personalized and precise treatment. | NCT06332716 |
|  |  | Q-GAIN (Using QPOP to Predict Treatment for Gastrointestinal cancer) | Drug screening in gastrointestinal cancer PDOs utilizing the QPOP platform for identification of novel effective drug combinations. | NCT04611035 |
|  | Glioblastoma | Glioma Stem Cell Organoids: Preclinical Model of Glioblastoma Heterogeneity to Explore Resistance Mechanisms to Conventional Treatment Schedules | Investigation of the mechanisms associated with the aggressive tumor growth of glioblastomas and seek for novel therapies able to overcome treatment resistance. | NCT04868396 |
| **Organoids** | Glioblastoma | Grafts of Patient-derived Glioblastoma Stem Cells onto Autologous Brain Organoids. A Precision Medicine Model for Testing Drugs Against Tumor Invasion | Brain organoids generated from iPSCs and coculture of glioma stem-like cells with iPSCs for drug screening of novel therapeutical agents able to inhibit invasion and restore ciliogenesis. | NCT05772741 |
|  |  | Pilot Trial for Treatment of Recurrent Glioblastoma | PDOs for drug screening and evaluation of drug response. | NCT05432518 |
|  |  | Modulation of Ciliogenesis in Glioma Stem Cells | Glioblastoma brain organoids culture to modulate cilium-related genes and administration of cilium-targeted drugs. | NCT05772767 |
|  | Glioma | The Role of B7-H4 in Tumor Vaccine | Experiments with PDOs and animal models to study the role of macrophage-derived B7-H4 in secretion of chemokines and treatment resistance of vaccines. | NCT06156150 |
|  |  | HiLoGlio Organoid Study: 'A Living Tissue Bank of PDOs From Glioma Tumors' | Development of new therapeutical strategies, and identification of malignant behavior and treatment resistance mechanisms utilizing glioma PDOs as disease models. | NCT04865315 |
|  |  | PTCs-based Precision Treatment Strategy on Recurrent High-grade Gliomas | Organoids culture derived from patient-derived tumor-like cell clusters (PTCs) for drug screening. | NCT05473923 |
|  |  | Evaluation of *ex vivo* Drug Combination Optimization Platform in Recurrent High Grade Astrocytic Glioma | Drug sensitivity tests using PDOs since their TME resemblance of high-grade astrocytic gliomas. | NCT05532397 |
|  | Gynecological Tumors | Construction of Gynecological Tumor Organoids | Protocol optimization for gynecological organoids and construction of a Biobank. | NCT06155370 |
|  | Head and Neck Squamous cell carcinoma (HNSCC) | SOTO Study: Prospective Study to Correlate the Treatment Sensitivity of PDOs With Clinical Outcomes in HNSCC Patients | Assessment of treatment response of HNSCC PDOs with the perspective to predict the response of the patients to the treatment. | NCT05400239 |
|  | Head and Neck cancer | Establishment of Squamous Cell Organoids of the Head and Neck to Assess Their Response to Innovative Therapies | Development of squamous cell PDOs for drug screening and assessment to patient specific novel therapies. | NCT04261192 |
|  |  | Immune Biomarker Study for Head and Neck Cancer | PDOs for functional biological analyses. | NCT05375266 |
|  | HCC | HCC Organoids to Explore TME and Test Treatment Efficacy - HCC Liver Organoids | Liver PDOs to study disease specific patterns and *in vitro* therapeutic response. | NCT06355700 |
| **Organoids** | HCC | Development of a Neuronal Microscope | PDOs for molecular characterization, functional morphological analysis and omics studies. | NCT06311396 |
|  |  | Prospective, Multicenter HCCIS Evaluation Study | HCC PDOs to test the effect of CD8+IL-33+ effector-memory cells as a translational approach. | NCT02718235 |
|  | HCC and CRC metastases | “Next Generation“ of Liver Derived-organoid Biobank: Case of CRC Metastases and HCC | Biobank buildout of liver PDOs (from liver metastases of CRC, hepatocellular adenoma, adenocarcinoma) in the prospect of using it for drug screening. | NCT05384184 |
|  | Intrahepatic cholangiocarcinoma | Clinical Transformation of Organoid Model to Predict the Efficacy of GC in the Treatment of Intrahepatic Cholangiocarcinoma | Personalized drug screening and evaluation of drug resistance in intrahepatic cholangiocarcinoma PDOs. | NCT05644743 |
|  | Kidney cancer | To Establish a Reproducible Organoid Culture Model with Human Kidney Cancer | Personalized medicine and novel therapies development using kidney PDOs. | NCT04342286 |
|  |  | Overtaking Intra and Inter Tumoral Heterogeneity In Von Hippel-Lindau Related Renal Cancer | Representative of intra- and inter-tumor heterogeneity PDOs to test antitumor agents, such as HIF2alpha inhibitor. | NCT06195150 |
|  | Liver, biliary and pancreatic cancers | A Study Designed to Develop *in vitro* Models of Liver, Biliary and Pancreatic Cancer for the Investigation of Tumor Biology and Potential Therapies | Assessment of molecular and cellular biology of liver, biliary and pancreatic PDOs for model creation. | NCT02436564 |
|  | Liver cancer | PDOs, PDOs-tumor-infiltrating Lymphocyte Coculture System, and Patient-derived Organotypic Tissue Spheroids for Drug Screen | Drug screening and personalized medicine investigation through lymphocyte coculture PDOs and patient-derived organotypic tissue spheroids. | NCT05913141 |
|  | Liver metastasis | Advanced Therapies for Liver Metastases | PDOs development for immune, gene and cell therapy approaches to treat liver metastasis derived from PDAC and CRC. | NCT04622423 |
|  | LC | Prospective Primary Human LC Organoids to Predict Treatment Response | Local irradiation of organoids and PDXs originated from surgical removed LC tissue. | NCT04859166 |
|  |  | LC Organoids and Patient Derived Tumor Xenografts | Development of lung PDOs and PDXs for disease modeling. | NCT05092009 |
|  |  | Generation of Lung Tumor Organoids from Patients Undergoing Diagnostic Bronchoscopy | Novel protocol for lung PDOs via diagnostic bronchoscopy and assessment of specific molecular pathways. | NCT06239584 |
| **Organoids** | LC | PDOs of Lung Cancer to Test Drug Response | Lung PDOs generation for disease modeling and personalized medicine (tests to the chemoresponse of organoids). | NCT03979170 |
|  |  | A Single-arm, Single-center Clinical Trial of Patient-derived LC Organoids for Predicting Therapeutic Response in Patients with Multiline Drug-resistant LC | Lung PDOs formation for drug screening and personalized medicine. Discovery of novel treatment to overcome multi-standard treatments resistance. | NCT05669586 |
|  |  | Detection of Genetic Markers of LC | PDOs undergoing resection for treatment of LC and mesothelioma. | NCT00280202 |
|  |  | Definition of the Status of the Human Lung Stem Cell Niches *ex vivo* in Tissue Biopsies Performed in Patients with Emphysema and Interstitial Fibrosis | PDOs for drug screening and development of patient personalized medicine. | NCT02705144 |
|  |  | PDO Model and Circulating Tumor Cells for Treatment Response of LC | The potential of PDOs to recapitulate patient responses to chemotherapeutic and targeted agents. | NCT03655015 |
|  | LC and others solid tumors | TCR-T Cell Immunotherapy of LC and Other Solid Tumors | PDOs coculture to screen anti-tumor T cells and to select monoclonal T cells for T cells receptor (TCR) cloning. | NCT03778814 |
|  | Meningioma | Establishment and Characterization of Meningioma PDOs | Establish and characterization of meningioma PDOs. | NCT04478877 |
|  | Mesothelioma | New Preclinical and Clinical Approaches to Mesothelioma | Test inhibitors on spheroids and organoids to assess the response of individual patients to therapy. | NCT06536179 |
|  | Metastatic Castration-resistant PC (mCRPC) | A Prospective, Open-label, Single-arm Study on the Efficacy and Safety of Organoid-Based Drug Sensitivity Screening to Guide the Treatment of mCRPC Patients with Bone Metastasis Progressed After First-line Treatment | Development of mCRPC with bone metastases PDOs for drug screening. | NCT06529549 |
|  | Metastatic solid cancers | High Dose Vitamin C Intravenous Infusion in Patients with Resettable or Metastatic Solid Tumor Malignancies | PDOs treated with vitamin C were added in culture medium to examine tumor response. | NCT03146962 |
|  |  | CPCT-05 Biopsy Protocol Patient Selection | PDOs culture for biomarker discovery. | NCT01904916 |
| **Organoids** | Nephroblastoma (Wilms tumor) | Treatment of Newly Diagnosed Patient's with Wilm's Tumor Requiring Abdominal Radiation Delivered with Proton Beam Irradiation | PDOs and PDXs to study the biology of Wilms tumor and compare responses to chemotherapeutic agents. | NCT04968990 |
|  | Neuroendocrine carcinomas | Cisplatinum and Everolimus in Patients with Metastatic or Unresectable NEC of Extrapulmonary Origin | PDOs formation upon a pre-treatment, and optional post-treatment to evaluate treatment response. | NCT02695459 |
|  |  | Single-cell Sequencing and Establishment of Cell Lines, Organoids, and Transplanted Tumor Models in Neuroendocrine Neoplasm | Gastrointestinal pancreatic neuroendocrine tumors PDOs establish as a research system to find novel molecular mechanisms and potential intervention targets. | NCT04927611 |
|  | NSCLC | Using *ex vivo* Tumoroids to Predict Immunotherapy Response In NSCLC | PDOs to predict immunotherapy responses in the context of patients with advanced/metastatic NSCLC receiving immune checkpoint inhibitors. | NCT05332925 |
|  |  | The Safety and Feasibility of Costal Bone Marrow Aspiration During Thoracic Surgery | Preclinical *ex/in vivo* treatment assessment in PDOs and PDXs. | NCT05251805 |
|  |  | Evaluation of Programmed Death Ligand 1 (PDL1) Response to Treatment in PDOs and Immune-marker Positron Emission Tomography (PET) Scanning in NSCLC | Evaluation of the response to Dostarlimab and Pembrolizumab in PDOs. | NCT06405230 |
|  |  | Evaluation of the Response to Tyrosine Kinase Inhibitors in Localized NSCLC Patients with EGFR Mutation in a PDO Model | Analysis of the *in vitro* efficacy of osimertinib in a PDO model alone or in combination. | NCT05136014 |
|  |  | PDO Drug Sensitivity Guided Treatment for Drug-resistant Recurrent NSCLC | PDOs for drug screening for patient guiding treatment. | NCT06406608 |
|  |  | The Correlation Study of the Drug Sensitivity Between *ex vivo* Model of NSCLC PDOs and Clinical Response in NSCLC Patients | Evaluation of NSCLC PDOs capacity to predict clinical response of target therapy or chemotherapy drugs. | NCT03453307 |
|  | OC | Translational Analysis in Longitudinal Series of OC ORganoids | Drug screening in high-grade serous OC PDOs. | NCT04555473 |
|  |  | Study of the Role of the TME in OC | Generation of OC PDOs to characterize *in vitro* interactions and molecular pathway among tumor cells, immune cells, and resident microbiota. | NCT06272240 |
| **Organoids** | OC | PDOs Immune Cells Coculture in OC: the Role of TME | Personalized medicine in OC PDOs coculture immune cells. | NCT06085404 |
|  |  | Study on the Consistency Evaluation of Organoids Used in the Clinical Treatment of OC With Anti-tumor Drugs | Anticancer drugs clinical efficacy prediction utilizing OC PDOs. | NCT05175326 |
|  |  | Drug Screening of PDOs From OC Culture to Personalized Therapy, an Exploratory Research | Validated OC PDOs, through NGS and immunohistochemistry, used for drug screening and personalized medicine. | NCT04768270 |
|  |  | Development and Validation of Growth Prediction Model for OC Organoids Based on Bright Field Image and Deep Learning | Prediction of OC PDOs growth leveraging artificial intelligence. | NCT06317610 |
|  |  | PDOs as a Promising Tool to Tailor OC Therapies (PANDORA) | Pursue of new therapies and drug development using OC PDOs. Correlation between PDOs therapies response and the treatment sensibility measured in the patients. | NCT06229522 |
|  |  | Longitudinal Sample Collection to Investigate Adaptation and Evolution of Ovarian High-grade Serous Carcinoma | Establishment of organoid cultures from ascites/peritoneal washings to study OC progression. | NCT05537844 |
|  |  | Drug Response in PDOs Models of Advanced or Recurrent OC, an Exploratory Research | Precise drug screening in OC PDOs and innovate drug research and development. | NCT05290961 |
|  |  | Individualized Precision Treatment Based on OC Organoid Model | Development of OC PDOs models for individualized drug screening. | NCT05813509 |
|  | Osteosarcoma | An Organoid-based Functional Precision Medicine Trial in Osteosarcoma: PREMOST | Drug screening in osteosarcoma PDOs models. | NCT06064682 |
|  |  | PDX Modeling to Test Drug Response for High-grade Osteosarcoma | PDOs for *in vitro* drug screening and *in vivo* PDXs drug testing. | NCT03358628 |
|  | PDAC | Innovative Therapeutic Treatments to Inhibit Perineural Invasion in Pancreatic Adenocarcinoma | PDAC PDOs and PDXs generation and evaluation of the effectiveness of NP-loaded hydrogel carrying FGFBP1 inhibitors and developed by CNR Nanotec Lecce. | NCT06616688 |
|  |  | Pharmacotyping of Patient-derived Pancreatic Cancer Organoids from Endoscopic Ultrasound-guided Biopsy as a Tool for Predicting Oncological Response | Drug screening in PDAC PDOs and comparison with patient’s response. | NCT05196334 |
| **Organoids** | PDAC | Drug Screening of Pancreatic Cancer Organoids Developed From EUS-FNA Guided Biopsy Tissues | Drug screening in PDAC PDOs. | NCT03544255 |
|  |  | PDO Generation in Pancreatic Cancer: a Single Centre, Open-label, Single Arm Feasibility Study | Drug screening and implementation of PDAC PDOs in clinical routine. | NCT05351983 |
|  |  | Establishing Organoids from Metastatic Pancreatic Cancer Patients, the OPT-I Study | Creation of PDAC PDOs and PDXs for personalized medicine through DNA/RNA profile analyses and delineation of disease pathological characteristics. | NCT03500068 |
|  |  | Development of a Prediction Platform for Neoadjuvant Treatment and Prognosis in Pancreatic Cancer Using *ex vivo* Analysis of Organoid Culture | Generation of new neoadjuvant chemotherapies before surgery employing PDAC PDOs and genomic analysis of each organoid. | NCT04777604 |
|  |  | Development of a Prediction Platform for Adjuvant Treatment and Prognosis in Pancreatic Cancer Using *ex v*ivo Analysis of Organoid Culture | Investigation of new adjuvant chemotherapies after surgery employing PDAC PDOs. | NCT04736043 |
|  |  | Prospectively Defining Metastatic Pancreatic Ductal Adenocarcinoma Subtypes by Comprehensive Genomic Analysis | Establishing PDOs to study novel therapies to treat pancreatic cancer. | NCT02869802 |
|  |  | PaTcH Study: A Phase 2 Study of Trametinib and Hydroxychloroquine in Patients with Metastatic Refractory Pancreatic Cancer | Development of PDOs to study the effects of anticancer drugs (trametinib combined with hydroxychloroquine) to overcome drug resistance. | NCT05518110 |
|  |  | A Prospective, Randomized, Controlled Trial of Chemotherapy for Advanced Pancreatic Cancer Based on Organoid Drug Sensitivity Test | PDAC PDOs formation as a tool to evaluate the consistency between the drug sensitivity and radiosensitivity test results and the treatment response of patients. | NCT04931381 |
|  |  | A Prospective, Randomized, Controlled Trial of Adjuvant Chemotherapy for Pancreatic Cancer Based on Organoid Drug Sensitivity Test | PDAC PDOs formation as a tool to evaluate the consistency between the drug sensitivity (adjuvant chemotherapy regimens) and radiosensitivity test results and the treatment response of patients. | NCT04931394 |
|  |  | Atezolizumab + Cabozantinib in Patients w/ Metastatic, Refractory Pancreatic Cancer | Preclinical studies of PDOs treatment with cabozantinib combined with atezolizumab. | NCT04820179 |
|  |  | Pancreatic Adenocarcinoma Signature Stratification for Treatment | PDOs models for personalized medicine in PDAC. | NCT04469556 |
| **Organoids** | PDAC | Volatile Organic Compound Assessment in Pancreatic Ductal Adenocarcinoma | PDOs to investigate the mechanisms of volatile organic compound production in PDAC. | NCT05727020 |
|  |  | Single-centre Phase 2 Open Label Two Independent Arms Study Assessing Organoid-driven Tumoral Chemosensitivity in Metastatic Pancreatic Cancer | Drug screening and personalized medicine using PDAC PDOs as disease models. | NCT06615830 |
|  |  | Province of Ontario Strategy for Personalized Management of Pancreatic Cancer Trial | Correlation between drug sensitivities in PDOs and molecular information from high-throughput drug screening. | NCT05927298 |
|  |  | Evaluation and Comparison of the Growth Rate of PDAC PDOs Generated from Matched Fine Needle Aspirations (FNA) and Fine Needle Biopsies (FNB) | Comparison of PDAC PDOs growth generated from matched FNA and FNB. | NCT03990675 |
|  |  | Simultaneous Establishment of Pancreas Cancer and Cancer-associated Fibroblast Using Endoscopic Ultrasound-guided fine needle Biopsy (EUS-FNB) Samples | Establishment of coculture PDAC and CAFs PDOs obtained from EUS-FNB samples. | NCT05571956 |
|  |  | A Prospective, Exploratory Study Evaluating the Effectiveness of Treatment Regimens for Locally Advanced/Metastatic Non-resectable Pancreatic Neuroendocrine Tumor Guided by *in vitro* Drug Sensitivity Testing of Tumor Organoids | Development of PDAC PDOs for drug screening and translation to the clinic. | NCT06246630 |
|  |  | EUS-guided Biopsy of Pancreatic Mass Lesions for Developing Patient-Derived Cancer Models | Development of new PDAC PDOs models through small biopsies (EUS-guided biopsies). | NCT03140592 |
|  | PDAC and Gastric cancer | A Multi-center, Prospective, Exploratory Study Evaluating the Effectiveness of Treatment Regimens for Metastatic Pancreatic and Gastric Cancer Guided by *in vitro* Drug Sensitivity Testing of Tumor Organoids | Comparison of the efficiency of drug treatments between metastatic PDAC and Gastric cancer PDOs and patients. | NCT05842187 |
|  | Pediatric cancers | Engineering Immune Organoids to Study Pediatric Cancer | Engineering immune organoids from pediatric patient tissues using iPSCs. | NCT05890781 |
| **Organoids** | Perihilar Cholangiocarcinoma | Stereotactic Body Radiation Therapy for Unresectable Perihilar Cholangiocarcinoma | Development of PDOs for radiotherapy response. | NCT03307538 |
|  | Peritoneal Carcinomatosis | PITCHER (Peritoneal Carcinomatosis Heterogeneity) | Organoid cultures and animal models for exome sequencing, transcriptome and methylome analysis. | NCT04714957 |
|  | Peritoneal Carcinomatosis from CRC | Organoids From Colorectal Peritoneal Metastases to Improve Cytoreductive Surgery and Patient-tailored Hyperthermic Intraperitoneal Chemotherapy (HIPEC) | Evaluation of HIPEC effect in peritoneal metastases from CRC PDOs. | NCT06057298 |
|  | Peritoneal Carcinomatosis from CRC and gastric cancer | Response Prediction of Hyperthermic Intraperitoneal Chemotherapy in Gastro- Intestinal Cancer | Assessment to genetic alterations in gastric or CRC with peritoneal carcinomatosis PDOs after drug screening analysis. | NCT05652348 |
|  | Pleural and peritoneal mesotheliomas | Immune Microenvironment and Gene Expression Profiling in Mesothelioma | PDOs/PDXs to evaluate pleural and peritoneal mesotheliomas tumorigenesis. | NCT06581549 |
|  | PC | Development of the Organoids Technique from Metastases from Patients with Advanced Form of PC: Use in Basic Research | Development of prostate PDOs from metastases to disease modeling and seek of new therapies. | NCT03952793 |
|  |  | INSIDE: Identification of Genomic Screening Pathways in Cancer Patients with DNA Repair Alterations | PDOs and PDXs for preclinical studies. | NCT06334809 |
|  |  | Combining Multi-omics Analysis and Organoid Models to Search for Novel Therapy Target in Metastatic PC | Metastatic PC PDOs used for analysis of their molecular characteristics and search of novel therapeutic targets. | NCT05577689 |
|  | Salivary Gland Carcinoma | Immune Biomarker Study for Salivary Gland Carcinoma | PDOs to analyze stated prognostic parameters. | NCT06047236 |
|  | Sarcoma | A Platform of PDXs and 2D/3D Cell Cultures of Soft Tissue Sarcomas (STS) | A platform of 2D/3D cell cultures, such as organoids, for the study of STS. | NCT02910895 |
| **Organoids** | Sarcoma or Melanoma | Using QPOP to Predict Treatment for Sarcomas and Melanomas | PDOs to evaluate drug response and sensitivity. | NCT04986748 |
|  | Small bowel carcinoid cancer | Natural History of Familial Carcinoid Tumor | Collection of tumor samples for histologic evaluation, culturing of intestinal organoids and genotyping. | NCT00646022 |
|  | Small cell LC | A Trial with Chemotherapy, Immunotherapy, and Radiotherapy for Patients with Newly Diagnosed Stage IV Small Cell LC | Study for patients with untreated Stage IV small cell LC. PDOs to evaluate the TME in those that respond to treatment vs those that do not respond | NCT04951115 |
|  |  | PDO Drug Sensitivity Guided Treatment for Recurrent Small Cell LC | Small Cell LC PDOs for the development of personalized treatment plans. | NCT06406660 |
|  | Solid tumors | Tumor Characterization to Guide Experimental Targeted Therapy - National | Experiments for understanding the biology of cancer (study of drug resistance mechanisms) and to identify potential targets for cancer patients (for drug screening). | NCT04723316 |
|  |  | Optimizing and Personalising Azacitidine Combination Therapy for Treating Solid Tumours QPOP and CURATE.AI | Generation of PDOs for drug selection, drug-drug interaction and drug response to improve personalized guidance. | NCT05381038 |
|  |  | The PIONEER Initiative: Precision Insights On N-of-1 *ex vivo* Effectiveness Research Based on Individual Tumor Ownership (Precision Oncology) | Establishment of PDOs for drug screening. | NCT03896958 |
|  |  | Personalized Models for Cancer Research | PDOs for personalized medicine in solid tumors. | NCT06350539 |
|  |  | KM3D Multicenter Cancer Consortium: Predicting Patient Response Using 3D Cell Culture Models | PDOs for drug tests and to predict patient responses to treatment. | NCT05338073 |
|  |  | Prospective Multicenter Study Evaluating Feasibility and Efficacy of Tumor Organoid-based Precision Medicine in Patients with Advanced Refractory Cancers | Generation of PDOs for chemotherapy, hormonal therapy and target therapy. | NCT05267912 |
|  |  | Selecting Chemotherapy with High-throughput Drug Screen Assay Using PDOs in Patients with Refractory Solid Tumors (SCORE) | Refractory solid tumors PDOs creation for drug screening and personalized medicine. | NCT04279509 |
| **Organoids** | Squamous cell carcinoma | Observational Study of Viral BIOmarkers and microRNAs in Tumors Orofarynx and Occult Tumors Positive for Papilloma Virus | PDOs and PDXs to identify new molecular drugs, which could solve resistance to radiochemotherapy. | NCT05918510 |
|  |  | Identification of the Pathogenetic Mechanisms Underlying Squamous Cell Carcinomas | Organoids coculture with CAFs derived from patients for experiments assessing the response to telomerase and deacetylase SIRT1 inhibitors. | NCT06236464 |
|  | Thyroid cancer | Efficacy of Organoid-Based Drug Screening to Guide Treatment for Locally Advanced Thyroid Cancer | Drug development and screening for personalized medicine using PDOs. | NCT06482086 |
|  | Undifferentiated cancer | Organoid Generation Study for Cancer | Assessment of cancer biology using PDOs models. | NCT05734963 |
|  |  | Development of Tumor Organoids from Undifferentiated Primary Carcinomas to Guide Therapeutic Decisions | Development of tumor organoids biobank. | NCT06612827 |
| **ToC** | Cervical and vaginal cancers | Primary Organoid Models and Combined Nucleic Acids Therapeutics for Anti-HPV Treatments | Organ-on-a-chip technology to screen innovative treatments for cervical and vaginal cancers. | NCT04278326 |
|  | CRC | Prospective Observation on the Accuracy of *in vitro* Screening of CRC Chemotherapy Drugs Based on Organoids-on-a-chip | Drug screening in ToC systems with CRC PDOs. | NCT04996355 |
|  | HCC | An Pancancer study on Organoid-on-chips technological system based on biopsy samples and its efficacy in predicting the response to mFOLFOX6 infusion in HCC | Evaluation of HCC PDOs (established in ToC systems) response to mFLOFOX6 infusion. | NCT05932836 |
|  | Liver cancer | Prognostic Value of Liver Cancer CTCs Isolated by a Novel Microfluidic Platform | PDOs and spheroids derived from isolated CTCs to obtain *in vitro* CTCs cell lines to reveal the metastatic mechanisms. | NCT05242237 |
|  | NSCLC | High Throughput Screening Device Based on 3D Nano-matrices and 3D Tumors with Functional Vascularization | Drug and immunotherapy screening device with vascularized PDOs. | NCT04826913 |
| **ToC** | PC | Observation of Clinical Consistency of Organoid-on-chips Drug Sensitivity Detection in Chemotherapy for PC Patients with Visceral Metastasis | Novel organoid chip model of organoids from PC to drug screening tests and personalized medicine. Potential exploratory study using organoid-immune coculture on chip to achieve new immune models. | NCT06536725 |

**References**

Ando, Y., Siegler, E. L., Ta, H. P., Cinay, G. E., Zhou, H., Gorrell, K. A., et al. (2019). Evaluating CAR‐T Cell Therapy in a Hypoxic 3D Tumor Model. *Adv Healthc Mater* 8. doi: 10.1002/adhm.201900001

Arena, S., Corti, G., Durinikova, E., Montone, M., Reilly, N. M., Russo, M., et al. (2020). A Subset of Colorectal Cancers with Cross-Sensitivity to Olaparib and Oxaliplatin. *Clinical Cancer Research* 26, 1372–1384. doi: 10.1158/1078-0432.CCR-19-2409

Ayuso, J. M., Truttschel, R., Gong, M. M., Humayun, M., Virumbrales-Munoz, M., Vitek, R., et al. (2019). Evaluating natural killer cell cytotoxicity against solid tumors using a microfluidic model. *Oncoimmunology* 8, 1553477. doi: 10.1080/2162402X.2018.1553477

Bayat, N., Izadpanah, R., Ebrahimi-Barough, S., Norouzi-Javidan, A., Ai, A., Mokhtari Ardakan, M. M., et al. (2018). The Anti-Angiogenic Effect of Atorvastatin in Glioblastoma Spheroids Tumor Cultured in Fibrin Gel: in 3D in Vitro Model. *Asian Pacific Journal of Cancer Prevention* 19, 2553–2560.

Camara, R., Ogbeni, D., Gerstmann, L., Ostovar, M., Hurer, E., Scott, M., et al. (2020). Discovery of novel small molecule inhibitors of S100P with in vitro anti-metastatic effects on pancreatic cancer cells. *Eur J Med Chem* 203, 112621. doi: 10.1016/j.ejmech.2020.112621

Chin, Y.-T., He, Z.-R., Chen, C.-L., Chu, H.-C., Ho, Y., Su, P.-Y., et al. (2019). Tetrac and NDAT Induce Anti-proliferation via Integrin αvβ3 in Colorectal Cancers With Different K-RAS Status. *Front Endocrinol (Lausanne)* 10. doi: 10.3389/fendo.2019.00130

Cho, K., Ro, S. W., Lee, H. W., Moon, H., Han, S., Kim, H. R., et al. (2021). YAP/TAZ Suppress Drug Penetration Into Hepatocellular Carcinoma Through Stromal Activation. *Hepatology* 74, 2605–2621. doi: 10.1002/hep.32000

de Witte, C. J., Espejo Valle-Inclan, J., Hami, N., Lõhmussaar, K., Kopper, O., Vreuls, C. P. H., et al. (2020). Patient-Derived Ovarian Cancer Organoids Mimic Clinical Response and Exhibit Heterogeneous Inter- and Intrapatient Drug Responses. *Cell Rep* 31, 107762. doi: 10.1016/j.celrep.2020.107762

Driehuis, E., Gracanin, A., Vries, R. G. J., Clevers, H., and Boj, S. F. (2020). Establishment of Pancreatic Organoids from Normal Tissue and Tumors. *STAR Protoc* 1, 100192. doi: 10.1016/j.xpro.2020.100192

El Harane, S., Zidi, B., El Harane, N., Krause, K.-H., Matthes, T., and Preynat-Seauve, O. (2023). Cancer Spheroids and Organoids as Novel Tools for Research and Therapy: State of the Art and Challenges to Guide Precision Medicine. *Cells* 12, 1001. doi: 10.3390/cells12071001

Escalona, R. M., Chu, S., Kadife, E., Kelly, J. K., Kannourakis, G., Findlay, J. K., et al. (2022). Knock down of TIMP-2 by siRNA and CRISPR/Cas9 mediates diverse cellular reprogramming of metastasis and chemosensitivity in ovarian cancer. *Cancer Cell Int* 22, 422. doi: 10.1186/s12935-022-02838-x

Jacob, F., Ming, G., and Song, H. (2020). Generation and biobanking of patient-derived glioblastoma organoids and their application in CAR T cell testing. *Nat Protoc* 15, 4000–4033. doi: 10.1038/s41596-020-0402-9

Jacob, F., Salinas, R. D., Zhang, D. Y., Nguyen, P. T. T., Schnoll, J. G., Wong, S. Z. H., et al. (2020). A Patient-Derived Glioblastoma Organoid Model and Biobank Recapitulates Inter- and Intra-tumoral Heterogeneity. *Cell* 180, 188-204.e22. doi: 10.1016/j.cell.2019.11.036

Kim, D., Choi, B., Ryoo, I., and Kwak, M.-K. (2018). High NRF2 level mediates cancer stem cell-like properties of aldehyde dehydrogenase (ALDH)-high ovarian cancer cells: inhibitory role of all-trans retinoic acid in ALDH/NRF2 signaling. *Cell Death Dis* 9, 896. doi: 10.1038/s41419-018-0903-4

Kim, M., Mun, H., Sung, C. O., Cho, E. J., Jeon, H.-J., Chun, S.-M., et al. (2019). Patient-derived lung cancer organoids as in vitro cancer models for therapeutic screening. *Nat Commun* 10, 3991. doi: 10.1038/s41467-019-11867-6

Kita, Y., Hamada, A., Saito, R., Teramoto, Y., Tanaka, R., Takano, K., et al. (2019). Systematic chemical screening identifies disulfiram as a repurposed drug that enhances sensitivity to cisplatin in bladder cancer: a summary of preclinical studies. *Br J Cancer* 121, 1027–1038. doi: 10.1038/s41416-019-0609-0

Koikawa, K., Kibe, S., Suizu, F., Sekino, N., Kim, N., Manz, T. D., et al. (2021). Targeting Pin1 renders pancreatic cancer eradicable by synergizing with immunochemotherapy. *Cell* 184, 4753-4771.e27. doi: 10.1016/j.cell.2021.07.020

Kondo, J., and Inoue, M. (2019). Application of Cancer Organoid Model for Drug Screening and Personalized Therapy. *Cells* 8, 470. doi: 10.3390/cells8050470

Kopper, O., de Witte, C. J., Lõhmussaar, K., Valle-Inclan, J. E., Hami, N., Kester, L., et al. (2019). An organoid platform for ovarian cancer captures intra- and interpatient heterogeneity. *Nat Med* 25, 838–849. doi: 10.1038/s41591-019-0422-6

Li, H., Harrison, E. B., Li, H., Hirabayashi, K., Chen, J., Li, Q.-X., et al. (2022). Targeting brain lesions of non-small cell lung cancer by enhancing CCL2-mediated CAR-T cell migration. *Nat Commun* 13, 2154. doi: 10.1038/s41467-022-29647-0

Maenhoudt, N., Defraye, C., Boretto, M., Jan, Z., Heremans, R., Boeckx, B., et al. (2020). Developing Organoids from Ovarian Cancer as Experimental and Preclinical Models. *Stem Cell Reports* 14, 717–729. doi: 10.1016/j.stemcr.2020.03.004

Maru, Y., Tanaka, N., Itami, M., and Hippo, Y. (2019). Efficient use of patient-derived organoids as a preclinical model for gynecologic tumors. *Gynecol Oncol* 154, 189–198. doi: 10.1016/j.ygyno.2019.05.005

Mazzocchi, A., Devarasetty, M., Herberg, S., Petty, W. J., Marini, F., Miller, L., et al. (2019). Pleural Effusion Aspirate for Use in 3D Lung Cancer Modeling and Chemotherapy Screening. *ACS Biomater Sci Eng* 5, 1937–1943. doi: 10.1021/acsbiomaterials.8b01356

Metastatic Prostate Cancer (2018). *New England Journal of Medicine* 378, 1653–1654. doi: 10.1056/NEJMc1803343

Millen, R., De Kort, W. W. B., Koomen, M., van Son, G. J. F., Gobits, R., Penning de Vries, B., et al. (2023). Patient-derived head and neck cancer organoids allow treatment stratification and serve as a tool for biomarker validation and identification. *Med* 4, 290-310.e12. doi: 10.1016/j.medj.2023.04.003

Mout, L., van Dessel, L. F., Kraan, J., de Jong, A. C., Neves, R. P. L., Erkens-Schulze, S., et al. (2021). Generating human prostate cancer organoids from leukapheresis enriched circulating tumour cells. *Eur J Cancer* 150, 179–189. doi: 10.1016/j.ejca.2021.03.023

Ramzy, G. M., Koessler, T., Ducrey, E., McKee, T., Ris, F., Buchs, N., et al. (2020). Patient-Derived In Vitro Models for Drug Discovery in Colorectal Carcinoma. *Cancers (Basel)* 12, 1423. doi: 10.3390/cancers12061423

Sachs, N., Papaspyropoulos, A., Zomer‐van Ommen, D. D., Heo, I., Böttinger, L., Klay, D., et al. (2019). Long‐term expanding human airway organoids for disease modeling. *EMBO J* 38. doi: 10.15252/embj.2018100300

Saito, Y. (2019). Establishment of an organoid bank of biliary tract and pancreatic cancers and its application for personalized therapy and future treatment. *J Gastroenterol Hepatol* 34, 1906–1910. doi: 10.1111/jgh.14773

Sayed, S., Sidorova, O. A., Hennig, A., Augsburg, M., Cortés Vesga, C. P., Abohawya, M., et al. (2022). Efficient Correction of Oncogenic *KRAS* and *TP53* Mutations through CRISPR Base Editing. *Cancer Res* 82, 3002–3015. doi: 10.1158/0008-5472.CAN-21-2519

Schnalzger, T. E., de Groot, M. H., Zhang, C., Mosa, M. H., Michels, B. E., Röder, J., et al. (2019). 3D model for <scp>CAR</scp> ‐mediated cytotoxicity using patient‐derived colorectal cancer organoids. *EMBO J* 38. doi: 10.15252/embj.2018100928

Smit, T., Calitz, C., Willers, C., Svitina, H., Hamman, J., Fey, S. J., et al. (2020). Characterization of an Alginate Encapsulated LS180 Spheroid Model for Anti-colorectal Cancer Compound Screening. *ACS Med Chem Lett* 11, 1014–1021. doi: 10.1021/acsmedchemlett.0c00076

Ukai, S., Honma, R., Sakamoto, N., Yamamoto, Y., Pham, Q. T., Harada, K., et al. (2020). Molecular biological analysis of 5-FU-resistant gastric cancer organoids; KHDRBS3 contributes to the attainment of features of cancer stem cell. *Oncogene* 39, 7265–7278. doi: 10.1038/s41388-020-01492-9

Vlachogiannis, G., Hedayat, S., Vatsiou, A., Jamin, Y., Fernández-Mateos, J., Khan, K., et al. (2018). Patient-derived organoids model treatment response of metastatic gastrointestinal cancers. *Science (1979)* 359, 920–926. doi: 10.1126/science.aao2774

Wang, Y., Jiang, T., Qin, Z., Jiang, J., Wang, Q., Yang, S., et al. (2019). HER2 exon 20 insertions in non-small-cell lung cancer are sensitive to the irreversible pan-HER receptor tyrosine kinase inhibitor pyrotinib. *Annals of Oncology* 30, 447–455. doi: 10.1093/annonc/mdy542

Wang, Y., Liao, H., Zheng, T., Wang, J., Guo, D., Lu, Z., et al. (2020). Conditionally reprogrammed colorectal cancer cells combined with mouse avatars identify synergy between EGFR and MEK or CDK4/6 inhibitors. *Am J Cancer Res* 10, 249–262.

Wei, X., Yang, J., Adair, S. J., Ozturk, H., Kuscu, C., Lee, K. Y., et al. (2020). Targeted CRISPR screening identifies PRMT5 as synthetic lethality combinatorial target with gemcitabine in pancreatic cancer cells. *Proceedings of the National Academy of Sciences* 117, 28068–28079. doi: 10.1073/pnas.2009899117

Wong, C.-W., Han, H.-W., Tien, Y.-W., and Hsu, S. (2019). Biomaterial substrate-derived compact cellular spheroids mimicking the behavior of pancreatic cancer and microenvironment. *Biomaterials* 213, 119202. doi: 10.1016/j.biomaterials.2019.05.013

Woo, D.-H., Chen, Q., Yang, T.-L. B., Glineburg, M. R., Hoge, C., Leu, N. A., et al. (2016). Enhancing a Wnt-Telomere Feedback Loop Restores Intestinal Stem Cell Function in a Human Organotypic Model of Dyskeratosis Congenita. *Cell Stem Cell* 19, 397–405. doi: 10.1016/j.stem.2016.05.024

Wu, Q., Wei, X., Pan, Y., Zou, Y., Hu, N., and Wang, P. (2018). Bionic 3D spheroids biosensor chips for high-throughput and dynamic drug screening. *Biomed Microdevices* 20, 82. doi: 10.1007/s10544-018-0329-x

Xie, G., Dong, H., Liang, Y., Ham, J. D., Rizwan, R., and Chen, J. (2020). CAR-NK cells: A promising cellular immunotherapy for cancer. *EBioMedicine* 59, 102975. doi: 10.1016/j.ebiom.2020.102975

Yang, G., Guan, W., Cao, Z., Guo, W., Xiong, G., Zhao, F., et al. (2021). Integrative Genomic Analysis of Gemcitabine Resistance in Pancreatic Cancer by Patient-derived Xenograft Models. *Clinical Cancer Research* 27, 3383–3396. doi: 10.1158/1078-0432.CCR-19-3975

Yao, Y., Xu, X., Yang, L., Zhu, J., Wan, J., Shen, L., et al. (2020). Patient-Derived Organoids Predict Chemoradiation Responses of Locally Advanced Rectal Cancer. *Cell Stem Cell* 26, 17-26.e6. doi: 10.1016/j.stem.2019.10.010

Yu, L., Li, Z., Mei, H., Li, W., Chen, D., Liu, L., et al. (2021). Patient‐derived organoids of bladder cancer recapitulate antigen expression profiles and serve as a personal evaluation model for CAR‐T cells *in vitro*. *Clin Transl Immunology* 10. doi: 10.1002/cti2.1248

Zhang, S., Iyer, S., Ran, H., Dolgalev, I., Gu, S., Wei, W., et al. (2021). Genetically Defined, Syngeneic Organoid Platform for Developing Combination Therapies for Ovarian Cancer. *Cancer Discov* 11, 362–383. doi: 10.1158/2159-8290.CD-20-0455

Zhou, Z., Van der Jeught, K., Fang, Y., Yu, T., Li, Y., Ao, Z., et al. (2021). An organoid-based screen for epigenetic inhibitors that stimulate antigen presentation and potentiate T-cell-mediated cytotoxicity. *Nat Biomed Eng* 5, 1320–1335. doi: 10.1038/s41551-021-00805-x
